# Foretinib can overcome common on-target resistance mutations after capmatinib/tepotinib treatment in NSCLCs with MET exon 14 skipping mutation

**DOI:** 10.1186/s13045-022-01299-z

**Published:** 2022-06-11

**Authors:** Toshio Fujino, Kenichi Suda, Takamasa Koga, Akira Hamada, Shuta Ohara, Masato Chiba, Masaki Shimoji, Toshiki Takemoto, Junichi Soh, Tetsuya Mitsudomi

**Affiliations:** Division of Thoracic Surgery, Department of Surgery, Kindai University Faculty of Medicine, 377-2 Ohno-Higashi, Osaka-Sayama, 589-8511 Japan

## Abstract

**Background:**

Capmatinib and tepotinib are guideline-recommended front-line treatments for non-small-cell lung cancer (NSCLC) patients with *MET* exon 14 skipping mutations (*METex14*). However, the emergence of acquired resistance to capmatinib/tepotinib is almost inevitable partially due to D1228X or Y1230X secondary mutations of the MET. In this study, we explored agents that are active against both D1228X and Y1230X MET to propose an ideal sequential treatment after capmatinib/tepotinib treatment failure in NSCLC patients with *METex14*.

**Methods:**

The inhibitory effects of 300 drugs, including 33 MET-TKIs, were screened in Ba/F3 cells carrying *METex14* plus *MET* D1228A/Y secondary mutations. The screen revealed four-candidate type II MET-TKIs (altiratinib, CEP-40783, foretinib and sitravatinib). Therefore, we performed further growth inhibitory assays using these four MET-TKIs plus cabozantinib and merestinib in Ba/F3 cells carrying *MET* D1228A/E/G/H/N/V/Y or Y1230C/D/H/N/S secondary mutations. We also performed analyses using Hs746t cell models carrying *METex14* (with mutant allele amplification) with/without D1228X or Y1230X in vitro and in vivo to confirm the findings. Furthermore, molecular dynamics (MD) simulations were carried out to examine differences in binding between type II MET-TKIs.

**Results:**

All 6 type II MET-TKIs were active against Y1230X secondary mutations. However, among these 6 agents, only foretinib showed potent activity against D1228X secondary mutations of the MET in the Ba/F3 cell and Hs746t in vitro model and Hs746t in vivo model, and CEP-40783 and altiratinib demonstrated some activity. MD analysis suggested that the long tail of foretinib plays an important role in binding D1228X MET through interaction with a residue at the solvent front (G1163). Tertiary G1163X mutations, together with L1195F/I and F1200I/L, occurred as acquired resistance mechanisms to the second-line treatment foretinib in Ba/F3 cell models.

**Conclusions:**

The type II MET-TKI foretinib may be an appropriate second-line treatment for NSCLCs carrying *METex14* after campatinib/tepotinib treatment failure by secondary mutations at residue D1228 or Y1230.

**Supplementary Information:**

The online version contains supplementary material available at 10.1186/s13045-022-01299-z.

## Background

Two type Ib MET-tyrosine kinase inhibitors (TKIs), capmatinib and tepotinib, were recently approved for the treatment of non-small-cell lung cancer (NSCLC) patients carrying *MET* exon 14 skipping mutations (*METex14*). These drugs yielded progression-free survival of 5.4–12.4 months in clinical trials [1, 2]; however, the emergence of acquired resistance is almost inevitable.

MET-TKIs are usually classified into two types based on their binding mode to MET [3]. Type I MET-TKIs bind to the ATP binding pocket of the active state of the MET (DFG-in) by interacting with the Y1230 residue [3]. Type I MET-TKIs are further subdivided into types Ia and Ib, according to their interaction with the G1163 residue (the solvent front residue). Crizotinib is a type Ia inhibitor, and capmatinib, tepotinib, and savolitinib are the type Ib inhibitors. Type Ib MET-TKIs are specific inhibitors of MET unlike type Ia TKI [4, 5]. Type II MET-TKIs are also ATP competitors; however, these inhibitors bind to the inactive state of the MET (DFG-out). Type II MET-TKIs include cabozantinib and merestinib, and these drugs have multi-kinase inhibitory activity.

Currently approved type Ib MET-TKIs, capmatinib and tepotinib, interact with the residue Y1230 residue; thus, it is reasonable that secondary mutations at Y1230 cause resistance to these drugs. In addition, through analyses of clinical specimens obtained from patients who acquired resistance to type I MET-TKIs, the emergence of secondary mutations at the D1228 position was also found to be frequent after capmatinib/tepotinib treatment failure [6–13].

As a potential second-line treatment, type II MET-TKIs that bind to the inactive form of MET have been suggested in previous studies to overcome many of the secondary mutations that emerge after front-line capmatinib/tepotinib monotherapy [14–17]. Although a few case studies have supported this hypothesis [6, 15], others have reported that patients who acquire D1228X secondary mutations may show a poor response to type II MET-TKIs [6, 14, 15, 18–23]. In addition, we and other groups have reported that cancer cells with D1228X showed higher IC_50_ values for type II MET-TKIs than cancer cells with Y1230X through in vitro experiments [15, 16, 24]. In this study, therefore, we aimed to identify the most effective type II MET-TKIs against resistant mutations to capmatinib/tepotinib, namely D1228X and Y1230X, through in vitro screening of all currently available type II MET-TKIs.

## Materials and methods

### Reagents

A total of 33 MET-TKIs and other receptor TKIs, multi-kinase inhibitors and cytotoxic inhibitors were evaluated in the present study. A SCADS inhibitor kit was provided by the Screening Committee of Anticancer Drugs supported by a Grant-in-Aid for Scientific Research on Innovative Areas, Scientific Support Programs for Cancer Research, from the Ministry of Education, Culture, Sports, Science, and Technology of Japan. In addition, we purchased almost all commercially available MET-TKIs which are under clinical development or were developed clinically in the past. The features of TKIs evaluated in the present study are summarized in the Additional file 1: Table S1. These drugs were dissolved in dimethyl sulfoxide (DMSO) (Sigma-Aldrich) and stored at − 80 °C until use.

### Cell lines

The interleukin-3 (IL-3) dependent murine pro-B cell line (Ba/F3) and myelomonocytic, macrophage-like, BALB/c mouse leukemia cells (WEHI-3) were provided by the RIKEN Bio Resource Center (Tsukuba, Japan). WEHI-3 secretes IL-3 into the medium and was thus used as a source of IL-3 for Ba/F3. The human gastric cancer-derived Hs746t cell line carrying *METex14* and *MET* amplification, was purchased from the American Type Culture Collection (Manassas, VA). Gp2-293 packaging cells were purchased from Takara (Kusatsu, Japan).

Ba/F3 cells were maintained in RPMI-1640 (Wako, Osaka, Japan) medium supplemented with 10% fetal bovine serum (FBS) (Sigma-Aldrich, St. Louis, MO,) and 1% penicillin–streptomycin (PS) (Wako, Osaka, Japan) at 37 °C in an incubator in a humidified atmosphere containing 5% CO_2_. Gp2-293 cells and Hs746t cells were maintained in Dulbecco’s modified Eagle’s medium (DMEM) containing 10% FBS and 1% PS at 37 °C in an incubator in a humidified atmosphere containing 5% and 10% CO_2_, respectively.

### Generation of Ba/F3 cells and Hs746 cells carrying METex14 plus secondary mutations

A Ba/F3 clone with *METex14* and MET-TKI-resistant Ba/F3 clones (*METex14* plus D1228A/E/G/H/N/Y or Y1230C/D/S/N/H) were established in our previous study [16]. These MET-TKI-resistant clones were established by MET-TKI exposure after ENU mutagenesis.

Transfection was applied to Ba/F3 parental cells and Hs746t cells to generate in vitro models of *METex14* plus one of the secondary mutations (D1228H/N/V/Y or Y1230H) as described previously [16]. Briefly, pRetroX IRES-ZsGreen1 carrying *METex14* was used as a template, and the retroviral vector constructs encoding *METex14* plus point mutations (D1228H/N/V/Y and Y1230H) were generated by a Prime STAR mutagenesis basal kit (Takara). All mutations were confirmed by direct sequencing. The viral particles were generated by co-transfection of these retroviral vector and the pVSV-G vector (Clontech) into Gp2-293 cells and were added to Ba/F3 and Hs746t cells. Ba/F3 cells and Hs746t cells infected with the retrovirus were collected using green fluorescence protein (GFP)-based fluorescence-activated cell sorting by a BD FACS Aria cell sorter (special order research product; BD Biosciences).

### Cell growth inhibition assay

A total of 3 × 10^3^ cells were seeded per well of 96-well plates. After 24 h, MET-TKIs were added at indicated concentrations. After a 72-h incubation, 10 μl of Cell Counting Kit-8 solution (Dojindo Laboratories, Kumamoto, Japan) was added to the wells, and the plates were incubated for an additional 3 h. The absorbance at 450 nm was read using a multiplate reader (Tecan, Mannedorf, Switzerland). The percentage of viable cells was evaluated and compared with that in DMSO-treated control samples. The half-maximal inhibitory concentration values (IC_50_) were determined by fitting a nonlinear regression curve to a variable slope model with normalized response in GraphPad Prism version 8 (GraphPad Software, San Diego, CA).

### Generation of foretinib-resistant clones by ENU mutagenesis

MET-TKI-resistant clones were generated by ENU mutagenesis as described previously [16]. Ba/F3 cells expressing MET mutations were exposed to 100 μg/ml ENU for 24 h. The cells were washed and cultured in RPMI containing 10% FBS for 24 h. Subsequently, 1 × 10^4^ cells were plated in 96-well plates in the presence of foretinib at indicated concentrations. The medium containing individual MET-TKIs was replaced once weekly for three weeks. When cell growth was detected macroscopically, total RNA was isolated from the resistant clones using an RNeasy Plus Mini kit (Qiagen, Hilden, Germany). Then, RNA was converted to cDNA using ReverTra Ace (TOYOBO, Osaka, Japan). The *MET* gene was sequenced from exon 15 to exon 21 using Genetic Analyzers 3130 or 3500XL (Applied Biosystems, Waltham, MA).

### Western blotting

Ba/F3 and Hs746t cells were treated with MET-TKIs at the indicated concentrations for 3 h. The cells were then washed twice with PBS and resuspended in lysis buffer. Lysates were quantified using a BCA protein assay (Bio-Rad, Hercules, CA), and proteins were electrophoresed and transferred to polyvinylidene difluoride membranes. Immunoblotting was performed with antibodies purchased from Cell Signaling Technology (Danvers, MA) according to the manufacturer’s instructions (Additional file 1: Table S2). Immunoblots were scanned using an Amersham Imager 680 (GE Healthcare, Chicago, IL).

### In vivo* analysis*

An in vivo study was performed at Kindai University in accordance with the guidelines of the Institutional Animal Care and Use Committee of Kindai University. Five-week-old nude male mice were purchased from CLEA Japan (Tokyo, Japan), and 1.0 × 10^6^ Hs746t cells were implanted in the right flank of each mouse. When the average tumor volume (length x width x width × 0.5) reached 0.75 cm^3^, the mice were randomized to three groups and treated once daily with foretinib, cabozantinib, and vehicle via oral gavage. Foretinib and cabozantinib were first dissolved in DMSO and then diluted with ddH2O to a dose of 16.36 mg/kg and 12.3 mg/kg, respectively. The final concentration of DMSO was 2%. The mixture of ddH2O containing 2% DMSO was used as a vehicle. Tumor size and body weight were monitored every two days and twice weekly, respectively, until the average tumor volume reached 2.0 cm^3^. Statistical analysis was performed using Kruskal–Wallis test with Dunnett’s multiple comparison test.

### Immunohistochemistry

Formalin-fixed, paraffin-embedded tissue Sects. (4-μm thick) obtained from in vivo experiments were deparaffinized and heated in Antigen Retrieval Citrate Solution, pH 6 (Dako, Glostrup, Denmark). The tissue sections were treated with Protein Block Serum-Free (Dako) and then incubated with a phospho-MET antibody (#3077) (Cell Signaling Technology) and then Histofine Simple Stain MAX PO(R) (Nichirei Bioscience, Tokyo, Japan). Peroxidase activity was visualized via the DAB reaction using a reagent from Dako. The sections were counterstained with hematoxylin.

### Molecular dynamics simulations

Using MolDesk Basic ver. 1.1.77 (IMSBIO, Tokyo, Japan), molecular dynamics (MD) simulations of MET-TKIs and the MET protein were performed. Co-crystal structures of wild type of MET kinase domain in complex with merestinib, foretinib, altiratinib (type II kinase inhibitors) or MET D1228V with foretinib were downloaded from The Protein Data Bank, respectively (PDB ID: 4eev, 6sd9, 5dg5 and 6sdc). The retrieved protein–ligand structures were processed by MolDesk Basic ver. 1.1.77 (IMSBIO, Tokyo, Japan) (1): addition of all hydrogens and deletion of all water molecules for protein. (2) Addition of all hydrogens and partial charge for ligand was performed using MOPAC7 AM1 BCC of MolDesk Basic. A docking pocket of each ligand for c-Met kinase domain was generated specifying the site where the MET inhibitor was originally bound. For foretinib and merestinib, the structurally determined ligands within 6sd9, 4eev were used. For cabozantinib, 3D structure file (ID: 25,102,847) was downloaded from PUBchem. Molecular docking simulation of foretinib, cabozantinib and merestinib with MET kinase domain (wild type or D1228V mutant) were carried out and docking score and RMSD were calculated using the sievgene of MolDesk Basic. Ligands (foretinib and merestinib) were used from co-crystal structure model within the above files. PyMOL (ver 2.5.2) was used to draw the 3D structure and to measure the distance between two points.

## Results

### D1228X as the most frequent on-target mutation causing resistance to type I MET-TKIs in the clinical settings

**Fig. 1:**
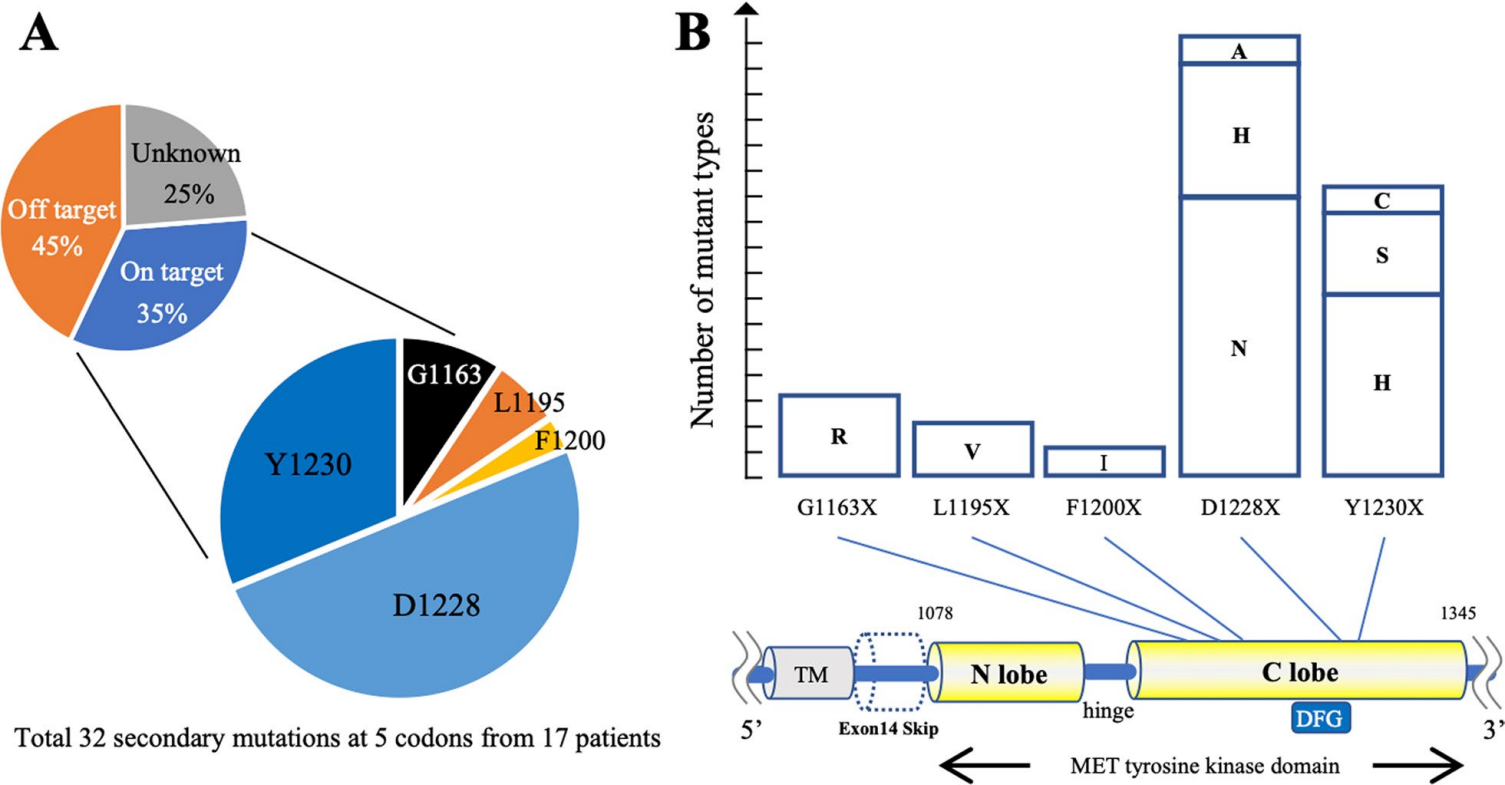
Summary of the acquired resistance mechanisms in MET-driven NSCLC patients in a clinical setting. **A, B** Summary of clinically reported acquired resistance mechanisms to MET-TKIs in *METex14*-positive lung cancer patients. The frequency of occurrence of on-target and off-target resistance mechanisms to MET-TKIs is based on previous data reported by Recondo G. et al. (Clin Cancer Res. 2019, reference no: 6). The details of secondary resistance mutations are summarized based on the previous literatures (reference no: 3–10). TM; Transmembrane domain, A; Alanine, C; Cysteine, D; Asparatic acid, F; Phenylalanine, G; Glycine, H; Histidine, I; Isoleucine, L; Leucine, N; Asparagine, R; Arginine, S; Serine, V; Valine, Y; Tyrosine

PubMed (https://pubmed.ncbi.nlm.nih.gov/) was used to review previous publications and summarize the on-target mechanisms of resistance to type I MET-TKIs in NSCLC patients carrying *METex14* [6–13]. Secondary mechanisms of resistance to crizotinib were predominantly reported prior to 2020 apparently due to easier clinical access to crizotinib. A total of 32 secondary mutations of 5 residues were demonstrated in 17 patients (Fig. 1A and B). D1228 and Y1230 were the common sites for secondary mutations conferring resistance to type I MET-TKIs, as we have reported previously [16].

We also reviewed the case reports of MET-driven mainly NSCLC patients who progressed on type I MET-TKIs, acquired secondary mutations at D1228 or Y1230, and were then treated by type II MET-TKIs, such as cabozantinib, merestinib, and glesatinib [6, 14, 15, 18–23]. The results of rebiopsy after type II MET-TKI treatment failure suggested that the Y1230X, but not D1228X, mutation could be eliminated by these type II TKIs (Table 1). These results suggested that second-line TKIs, which are active against both Y1230X and D1228X, are essential for effective overcoming of acquired resistance to capmatinib or tepotinib.

**Table 1:** Clinical cases who were treated with type I MET-TKI followed by type II MET-TKI

Case	Histology	Driver mutation	Prior type I MET-TKI	Best response	TTF (month)	MET secondary mutation	→	Switched type II MET-TKI	Best response	TTF (month)	MET secondary mutation	References
1	Ad	METex14	crizotinib	SD	15.9	Y1230C	→	merestinib	PR	N/A	N/A	[6]
2	NSCLC	KIF5B-MET	crizotinib	PR	NA	Y1230H	→	cabozanitnib	PR	N/A	N/A	[21]
3	Ad	EGFR mt	savolitinib	CR	9	D1228V	→	cabozantinib	PR	> 5	N/A	[14]
		+	+					+				
		MET amp	osimertinib					elrotinib				
4	Ad	CD74-ROS1	crizotinib	PR	10	D1228N	→	cabozantinib	PR	3	D1228N	[22]
5	Ad	EGFR mt	savolitinib	PR	18	D1228N	→	cabozantinib	SD	3	D1228N	[20]
		+	+			D1228Y		+			D1228Y	
		MET amp	osimertinib			D1228H		osimertinib			D1228H	
						Y1230C						
6	NSCLC	METex14	crizotinib	PR	NA	D1228N	→	cabozantinib	PD	(-)	N/A	[21]
7	Ad	METex14	crizotinib	PR	8	G1163R	→	glesatinib	PD	(-)	G1163R	[15]
						D1228N					L1195V	
						Y1230H					D1228N	
						Y1230S						
8	Ad	EGFR mt	crizotinib	PR	4	D1228N	→	cabozantinib	PD	(-)	D1228N	[18]
		+	+			D1228H		+				
		MET amp	osimertinib			Y1230H		osimertinib				
						D1231Y						
9	NSCLC	KIF5B-RET	capmatinib	SD	4.5	D1228N	→	cabozantinib	PD	(-)	D1228N	[23]
		+	+					+				
		MET amp	selpercatinib					selpercatinib				
10	Breast cancer	MET amp	crizotinib	PR	9	D1228N	→	cabozantinib	PD	(-)	N/A	[19]

*NSCLC* Non-small cell lung cancer, *Ad* Lung adenocarcinoma, *mt* mutation, *amp* amplification, *TTF* Time to treatment failure, *CR* Complete response, *PR* partial response, *SD* Stable disease, *PD* Progressive disease, *N/A* Not available

### Comprehensive screening to identify active drugs against D1228X or Y1230X secondary mutations

The D1228X mutations are potential refractory mutations against type II MET-TKIs that were used in our previous study [16]. Therefore, we first used Ba/F3 cells with *METex14* plus D1228A or D1228Y, which showed resistance to cabozantinib and merestinib in our previous study [16], to comprehensively screen the inhibitory effects of 300 anticancer drugs (100 nM), including 33 MET-TKIs and 48 other RTK inhibitors. As expected, all type I MET-TKIs were inactive against Ba/F3 cells with D1228A/Y mutations, while four type II MET-TKIs, namely altiratinib, CEP-40783, foretinib, and sitravatinib, inhibited growth by less than 50% compared to that in the DMSO-treated control. No other drugs showed MET-mutant cell-specific activity (Additional file 2: Fig. S1 and Additional file 1: Table S3).

We then expanded the cohort of Ba/F3 clones with secondary D1228X or Y1230X mutations to cover all clinically reported amino acid substitutions. Among them, Ba/F3 clone with D1228V was not obtained by ENU mutagenesis in our previous study [16]; therefore, we established a Ba/F3 clone with *METex14* plus D1228V by gene transfection in this study. Prior to subsequent analysis, the IC50 values of MET-TKIs obtained based on the data of the cell growth inhibition assay were compared in ENU-derived Ba/F3 cells with secondary mutations and vector-transfected Ba/F3 cells with secondary mutations to ensure that the results were comparable (Additional file 2: Fig. S2A–C).

**Fig. 2:**
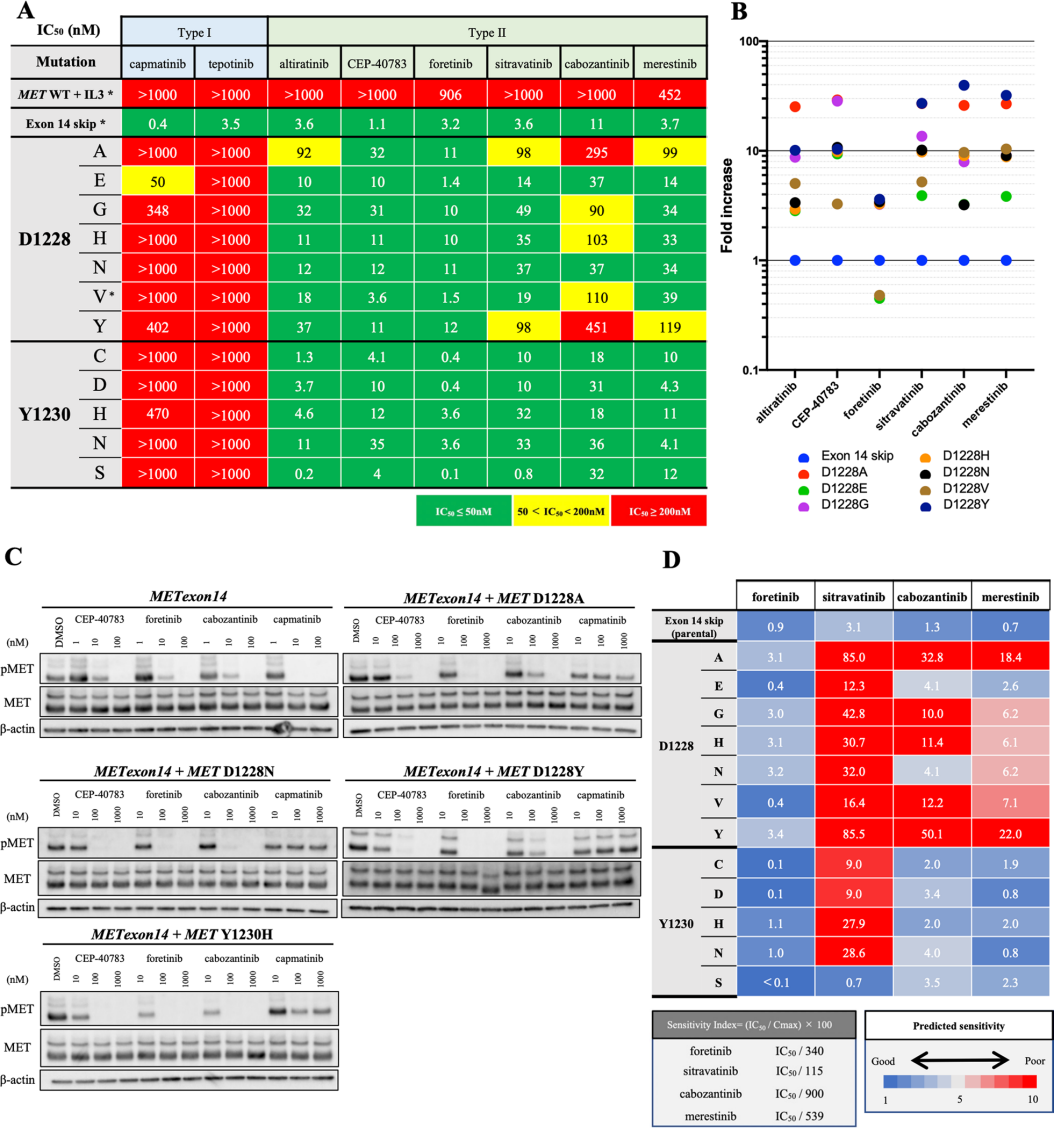
Evaluation of eight MET-TKIs, including four candidates, against clones with *MET* D1228X or Y1230X. **A** The IC_50_ values (nM) of each drug for Ba/F3 cells harboring *METex14* plus the indicated *MET* secondary mutations are expressed according to the indicated color scale. * indicates Ba/F3 clones generated using the corresponding vectors. Other cell lines were derived by ENU mutagenesis. Growth inhibition curves are summarized in Additional file 2: Fig. S2A and B. **B** The IC_50_ values of each drug for cells with each secondary mutation are expressed as fold increases of the IC_50_ for parental cells with *METex14* in dot plots. **C** Western blot analyses of Ba/F3 cells with *METex14* with/without secondary mutations treated with MET-TKIs at the indicated concentrations for 3 h. **D** The sensitivity index (SI) was defined as the IC_50_ value divided by the concentration max (*C*_max_) of each drug, which was reported in a clinical study. SI is expressed in a heatmap by the indicated color scales. For cabozantinib, since no PK data at 60 mg/day were available, the values were estimated from those reported at 80 mg/day and 40 mg/day based on previous reports (Kuzrock, R. et al. J Clin Oncol. 2011)

The detailed IC_50_ values in Ba/F3 cells with *METex14* plus D1228X or Y1230X (D1228A/E/G/H/N/V/Y or Y1230C/D/H/N/S) are summarized in Fig. 2A. In addition to the above four type II MET-TKIs (altiratinib, CEP-40783, foretinib and sitravatinib), we included merestinib and cabozantinib because these two agents are currently being evaluated in phase II clinical trials (Additional file 1: Table S4). We confirmed that capmatinib and tepotinib were inactive against all tested Ba/F3 cells. On the other hand, all six type II MET-TKIs showed potent activity against Ba/F3 cells with *METex14* plus Y1230X mutations (Additional file 2: Fig. S2). The inhibitory effects against cells with D1228X mutations were heterogeneous among type II MET-TKIs: foretinib and CEP-40783 were the most active. Through comparison of the IC_50_ values in cells with *METex14* plus D1228X vs. those with *METex14* alone, we observed that only foretinib had similar IC_50_ values in all Ba/F3 cells with secondary D1228X mutations (Fig. 2B). The effect of inhibition of MET phosphorylation was in accordance with the results of the growth inhibition assay (Fig. 2C). We also found that among the type II MET-TKIs, foretinib showed superior efficacy in terms of the sensitivity index, which accounted for both its IC_50_ values in Ba/F3 models and clinically achievable drug concentrations (Fig. 2D).

### In vitro activity of foretinib and other type II MET-TKIs in an Hs746t model

**Fig. 3:**
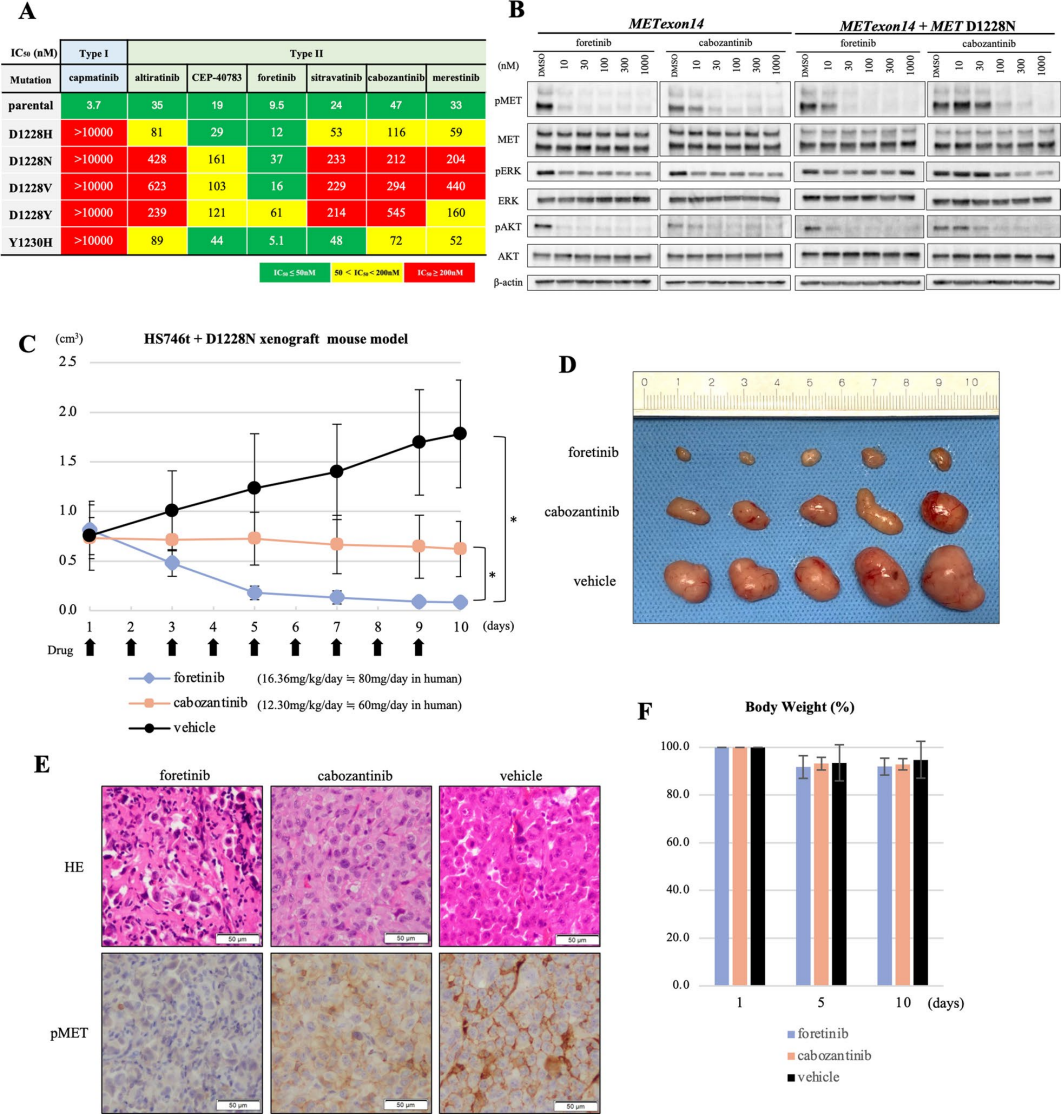
Evaluation of type II MET-TKIs in Hs746t in vitro and in vivo models. **A** The IC_50_ values (nM) of each drug for Hs746t cells harboring *METex14* plus the indicated *MET* secondary mutations are expressed according to the indicated color scale. Growth inhibition curves are summarized in Additional file 2: Fig. S3. **B** Western blot analyses of Hs746t cells with *METex14* with/without D1228N secondary mutation treated with MET-TKIs at the indicated concentrations for 3 h. *β*-actin was used as a loading control. **C** Changes in tumor size in vivo. Clinical dose of foretinib and cabozantinib used in phase II trial or clinical practice was converted to equivalent dose in mice considering the body surface area (Nair AB, Jacob S. J Basic Clin Pharm. 2016). Tumor size was measured every 2 days. Tumor Volume (TV) was calculated as follows: TV (cm^3^) = length × width × width × 0.5. Statistical analysis was performed using the Kruskal–Wallis test with the Dunnett’s multiple comparison test. *P* < 0.05 was considered to indicate statistical significance (* means *P* < 0.05). **D** Comparison of resected tumors treated with each drug from sacrificed mice on day 10. **E** H&E-stained histopathological images and phosphorylated MET in resected tumors treated with each drug determined by immunohistochemistry are shown. **F** The change (%) in the average body weight of mice during the treatment is shown compared to body weight before treatment

We also employed the Hs746t cell line, which originally harbored *METex14* and is sensitive to MET-TKIs. We introduced cDNA vectors with *METex14* plus D1228X (H/N/V/Y) or Y1230H secondary mutations into Hs746t cells to generate in vitro models with a secondary mutation in a part of the MET protein (with exon 14 skipping). We selected these four variants based on their frequencies in clinical samples (D1228N/H, Fig. 1B), the presence of a determined 3D structure (D1228V) and the potential lower efficacy of type II MET-TKIs in our previous study (D1228Y) [16]. Similar to the results in Ba/F3 models, foretinib was active against Hs746t cells with all D1228X or Y1230H secondary mutations tested (Fig. 3A and B and Additional file 2: Fig. S3). The IC_50_ values of cabozantinib and merestinib in Hs746t cells carrying the Y1230H secondary mutation were within a twofold increase compared with those in the parental cells. However, most of the IC_50_ values for cabozantinib and merestinib in Hs746t cells carrying D1228X secondary mutations were at least fivefold higher than those in the parental Hs746t cells. Western blot analysis revealed that foretinib, but not cabozantinib, inhibited the phosphorylation of MET, as well as the phosphorylation of AKT, in Hs746t cells with the D1228N secondary mutation in a dose-dependent manner (Fig. 3B).

### In vivo evaluation of the efficacy of foretinib versus cabozantinib

To confirm the in vitro findings, we established xenograft models using Hs746t cells with *METex14* plus the secondary mutation D1228N. The D1228N mutation was selected because this amino acid substitution was frequently detected in the clinical setting (Fig. 1B). After randomization, we treated mice with vehicle (control), foretinib, or cabozantinib orally. The drug dosages were determined based on a phase II clinical trial (80 mg/day) for foretinib and on the dosing used in clinical practice (60 mg/day) for cabozantinib [25–27]. As a result, we found that cabozantinib significantly inhibited tumor growth compared to that in the control; however, the mean tumor shrinkage rates remained within 20% of one another (Fig. 3C and D). On the other hand, in the foretinib group, all mice showed a major partial response of up to 90% shrinkage. By immunohistochemistry (IHC) analysis, we found that MET phosphorylation was almost completely diminished in tumors treated with foretinib; on the other hand, it remained weak in tumors treated with cabozantinib (Fig. 3E). There was no significant difference in body weight loss between the treatment arms (Fig. 3F).

### MD analysis of drug binding in MET with D1228X mutations

**Fig. 4:**
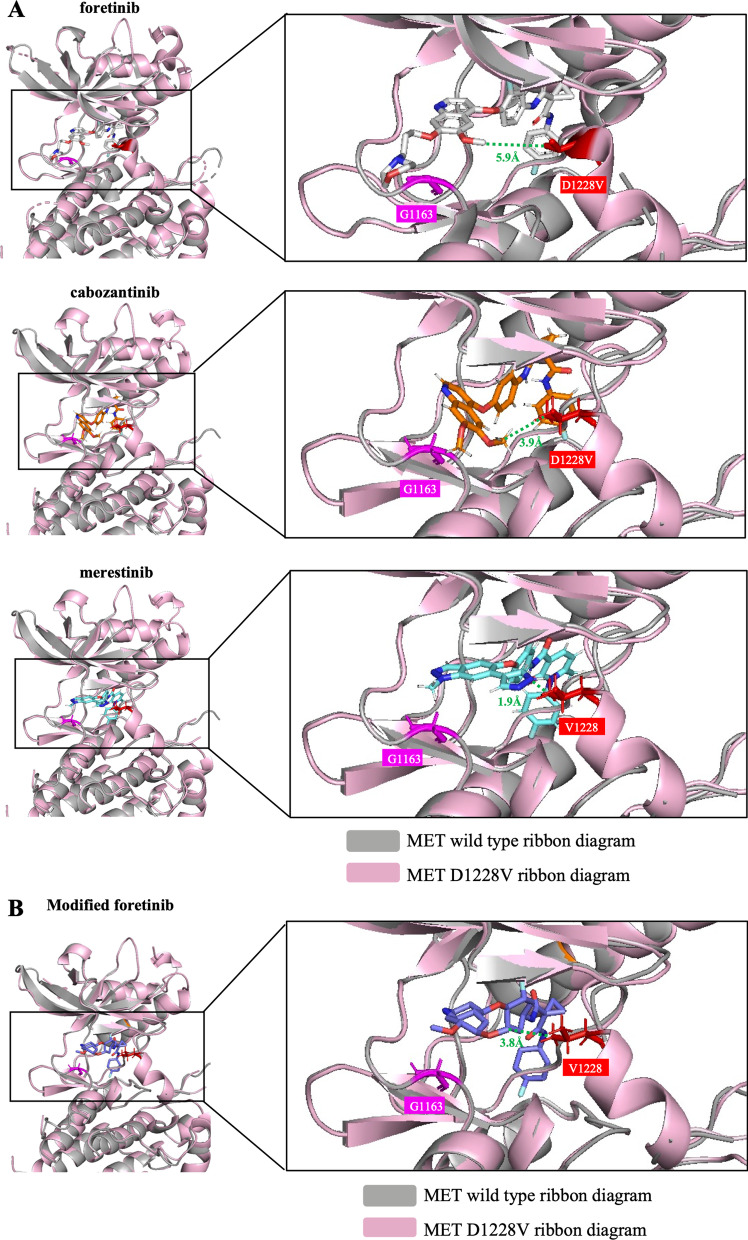
Comparison of the binding models of foretinib, cabozantinib and merestinib against MET with D1228V mutation. Using MolDesk Basic ver. 1.1.77, molecular docking simulations of foretinib, cabozantinib and merestinib with the c-Met kinase domain (wild type or D1228V mutant) were carried out. Using PyMOL (ver 2.5.2), the 3D docking model of each MET-TKI and wild-type MET was aligned to the structure of MET D1228V. **A** The closest distances (Å) between the quinoline or pyrazole group of cabozantinib, merestinib, or foretinib and MET V1228 were measured by PyMOL. **B** The closest distances between the quinoline group of modified foretinib and MET V1228 were measured by PyMOL

To investigate potential reasons for the higher sensitivity of foretinib compared with cabozantinib and merestinib to cancer cells harboring secondary D1228X mutations, we performed MD simulation analysis. Based on a previously determined 3D crystal structure of foretinib bound to MET with D1228V [28], we compared distances between foretinib or cabozantinib/merestinib and V1228. The smallest distances between the quinoline or pyrazole group of merestinib, cabozantinib and foretinib and MET V1228 were 1.9, 3.9 and 5.9 Å, respectively (Fig. 4A). We computationally replaced the long tail of foretinib with a small methyl group (producing a structure that resembles cabozantinib; the structures of these drugs are shown in Additional file 2: Fig. S6), and the results of MD analysis showed that the smallest distance between MET V1228 and this structure obtained by foretinib modification was reduced (5.9–3.8 Å) (Fig. 4B). Although the smallest distances between D1228 (wild type) and these drugs are not available, this result suggests that steric blockade is one of the potential reasons for the lower efficacy of cabozantinib/merestinib compared with that of foretinib in tumors with D1228X secondary mutations.

### Tertiary mutation at G1163, a residue at the solvent front, may cause resistance to foretinib

**Fig. 5:**
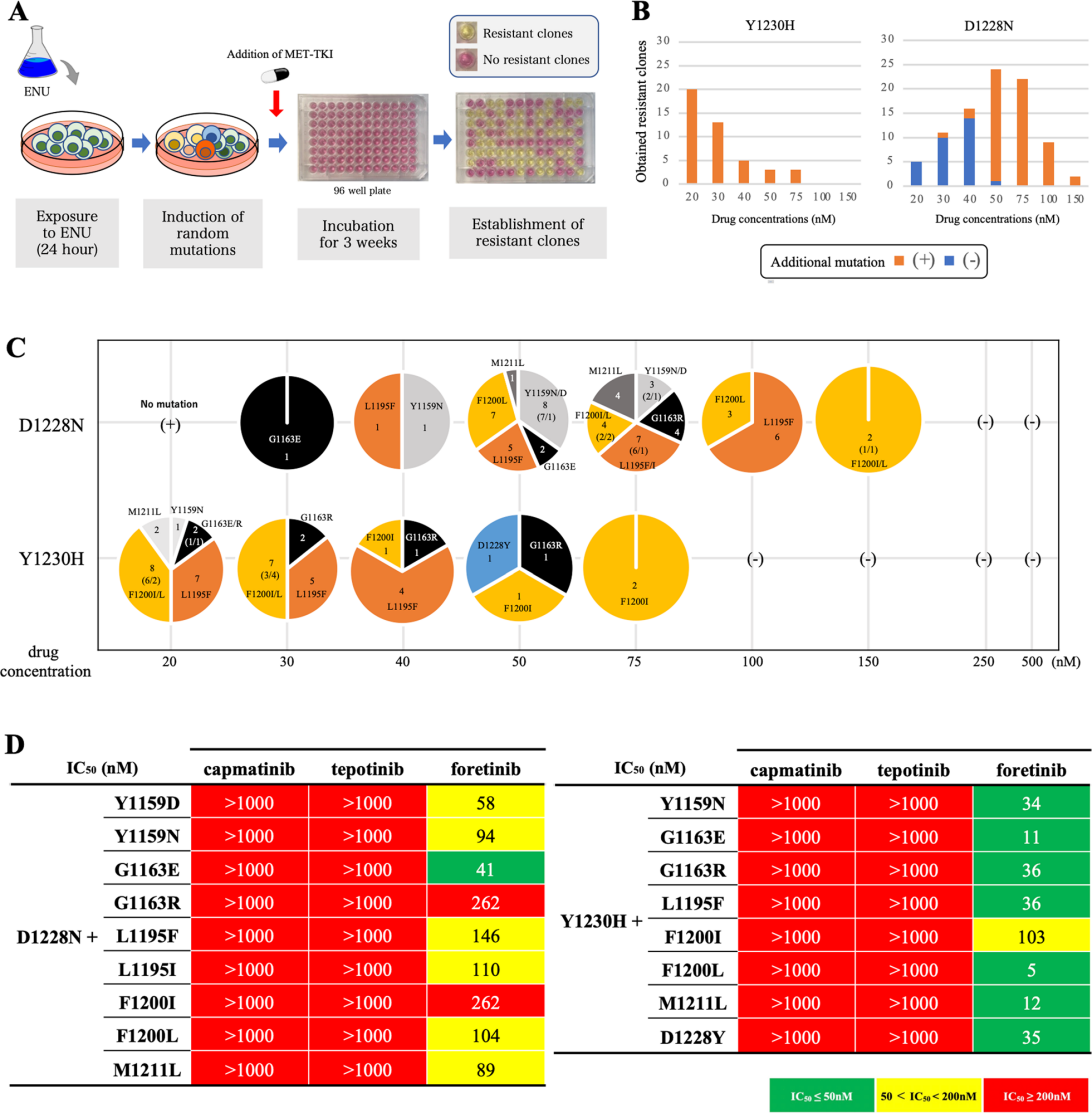
Analysis of the tertiary resistance mutations against second-line foretinib. **A** Schematic showing ENU mutagenesis. **B** The number of obtained resistant clones and the percentage of clones that carried tertiary mutations are shown for each drug concentration. Those with a tertiary mutation are shown in orange, and those without are shown in blue. **C** The detailed tertiary mutations obtained from ENU mutagenesis screening with foretinib are summarized for each drug concentration. **D** The MTT assay was performed with the obtained resistant clones with a tertiary resistant mutation, and IC_50_ values were determined. IC_50_ values are classified based on the indicated colors. They were also evaluated for the presence of cross-resistance to capmatinib and tepotinib. Growth inhibitory curves are summarized in Additional file 2: Fig. S7

We also explored residues that may cause resistance to the second-line treatment foretinib after the acquisition of D1228N or Y1230H secondary mutation through ENU mutagenesis screening (Fig. 5A). We found that G1163X, L1195F/I, and F1200I/L (and other minor mutations) caused resistance to the second-line foretinib treatment models (Fig. 5B–D and Additional file 2: Fig. S7A, B) especially in the models with the secondary D1228N mutation; on the other hand, these mutations did not confer high level of resistance to foretinib in the models with the secondary Y1230H mutation.

In our previous study, we observed that F1200I/L and L1195F are frequent resistance mutations against type II MET-TKIs such as cabozantinib/merestinib. To examine whether the frequent emergence of G1163X mutations at the solvent front is specific for foretinib, we also performed ENU mutagenesis analysis in the context of exposure to the front-line treatment foretinib. As expected, G1163X was a common secondary mutation observed in this setting (Additional file 2: Figs. S4B and S5). As shown in Fig. 4, the bound drug was located near the residue G1163 in the case of foretinib but not cabozantinib or merestinib. Therefore, it can be hypothesized that a change in the amino acid at this position may structurally block the binding of foretinib but not cabozantinib/merestinib.

## Discussion

The currently approved MET-TKIs capmatinib and tepotinib bind MET in an active state (DFG in conformation) at the Y1230 position using *π*–*π* stacking interactions. In addition, when MET is in an active state, the residue D1228 makes up the ATP binding pocket by engaging in a salt bridge with K1110 [3]. Therefore, it is reasonable that secondary mutations at these residues will cause acquired resistance to capmatinib and tepotinib. In this study, we explored adequate second-line MET-TKIs with high activity against secondary mutations that emerge after capmatinib or tepotinib treatment failure. We found that type II MET-TKIs show various activities against secondary D1228X mutations, although many of them were active against secondary Y1230X mutations. Similar to lorlatinib, which is active against a wide range of ALK secondary mutations [29, 30], second-line MET-TKIs are anticipated to overcome both D1228X and Y1230X mutations because of potential heterogeneity in secondary resistance mutations within individual patients [6, 31]. Through analyses in Ba/F3 cells, Hs746t cells, and in vivo models, we found that the drug foretinib can inhibit both D1228X and Y1230X secondary mutations.

As one of the reasons for the different efficacies against D1228X secondary mutations between foretinib and other type II MET-TKIs, in this study, we found that the distances between V1228 and foretinib were larger than the distance between V1228 and cabozantinib/merestinib (Fig. 4). In general, the 4-fluorophenylamine group of type II MET-TKIs enables binding of the drug to the deep extended hydrophobic pocket of the MET [3]. In the DFG-out conformation, to which type II MET-TKIs bind, the activation loop region, where both D1228 and Y1230 are located, are assumed to be disordered [28]. Indeed, the structure of the activation loop in the DFG-out conformation is often unresolved in deposited files of the wild-type MET structure (PDB ID: 4eev, 6sd9 and 3f82) from the Protein Data Bank, as shown in Additional file 2: Fig. S8A. However, Gavin et al. found that when a mutation occurs at D1228, the structure is fixed due to the formation of an alpha-helix in the activation loop, which reduces the size of the hydrophobic pocket, as shown in Additional file 2: Fig. S8B [28]. We suggest that this fixed conformation can facilitate the reduced activities of cabozantinib and merestinib. On the other hand, we found that secondary or tertiary mutations of residue G1163 at the solvent front cause resistance to foretinib. This is similar to the situation with crizotinib; G1163X mutations have been reported to cause crizotinib resistance [6, 7]. In the case of TKI treatment for NSCLC patients with ALK fusion, it has been reported that the interaction of the pyrazolopiperidine tail of crizotinib with G1202 at the solvent surface area is important for the appropriate binding of crizotinib to ALK [32]. In the docking model analysis, an interaction between the residue G1163 and the long tail of foretinib was observed. Therefore, the long tail of foretinib may also play an important role in binding to MET through interaction with the solvent surface area. The data of thorough MD analysis confirmed that the smallest distance between MET V1228 and modified foretinib, which lacks the long tail, was reduced (Fig. 4B). These results indicate that the long tail of foretinib provides stability in the binding of foretinib to MET at the solvent surface area and thus is less susceptible to the narrowing of the pocket by the formation of an alpha-helix.

The safety and tolerability of foretinib have been confirmed in two phase I trials (Additional file 1: Table S5) [33, 34]. However, the clinical development of foretinib has now been terminated due to the lower efficacy of this drug. We consider that the negative data from these phase II trials are partially due to inappropriate patient selection (Additional file 1: Table S6) [26, 27, 35–37]. Indeed, in a subgroup analysis of a phase II trial for papillary renal cell carcinoma, the objective response rate and disease control rate were 50% and 100%, respectively, for patients with the germline point mutations of *MET* [26]. Considering the current circumstances, we propose (1) re-evaluation of foretinib in the second-line setting for NSCLC patients who acquired resistance to capmatinib or tepotinib by secondary mutations or (2) development of a novel type II MET-TKI with a long tail that interacts with the residue G1163 of the MET.

## Conclusion

In this study, we explored adequate second-line MET-TKIs that show high activity against secondary mutations that emerge after treatment failure of two approved type Ib MET-TKIs, capmatinib and tepotinib. We showed that many type II MET-TKIs can overcome Y1230X mutations; however, our results suggest that cabozantinib and merestinib, which are currently under clinical development, may be insufficient to overcome resistance conferred by D1228X secondary mutations. On the other hand, foretinib showed potent activity against both D1228X and Y1230X secondary mutations.

## Supplementary Information

**Additional file 1:** **Table S1**. A list of tyrosine kinase inhibitors used in this study. **Table S2**. A list of antibodies used in this study. **Table S3.** Results of drug screening other than tyrosine kinase inhibitors. **Table S4.** Type II MET-TKIs evaluated in this study. **Table S5.** Clinical information of two phase I studies of foretinib. **Table S6**. Clinical information of five phase II studies of foretinib.

**Additional file 2**: **Fig. S1.** Drug screening against MET D1228A/Y using a drug library. **Fig. S2.** Summary of growth inhibitory curves of eight MET-TKIs for Ba/F3 cells with each MET mutation. **Fig. S3.** Evaluation of the effects of secondary mutations on sensitivity to MET-TKIs using the Hs746t cell line. **Fig. S4.** Analysis of the secondary resistance mutations against 1st line foretinib. **Fig. S5.** Growth inhibition curves for ENU-derived foretinib resistant Ba/F3 cells carrying MET secondary mutation. **Fig. S6.** Comparison of the structures of each type II MET-TKI. **Fig. S7.** Summary of growth inhibitory curves of three MET-TKIs for Ba/F3 cells with MET tertiary mutations. **Fig. S8.** Structure of MET in the DFG-in and DFG-out conformations.

## Data Availability

All data generated or analyzed during this study are included in this published article and its additional file.

## Abbreviations

NSCLC: Non-small-cell lung cancer
METex14: MET exon 14 skipping mutation
TKI: Tyrosine kinase inhibitor
MD simulation: Molecular dynamics simulation
