# Supplementary material for: Foretinib can overcome common on-target resistance mutations after capmatinib/tepotinib treatment in NSCLCs with MET exon 14 skipping mutation
Source: J Hematol Oncol. 2022 Jun 11;15:79. doi: 10.1186/s13045-022-01299-z (PMC9188708; doi:10.1186/s13045-022-01299-z)
Supplement: Supplementary file 1 — Additional file 1: Table S1. A list of tyrosine kinase inhibitors used in this study. Table S2. A list of antibodies used in this study. Table S3. Results of drug screening other than tyrosine kinase inhibitors. Table S4. Type II MET-TKIs evaluated in this study. Table S5. Clinical information of two phase I studies of foretinib. Table S6. Clinical information of five phase II studies of foretinib. [file 13045_2022_1299_MOESM1_ESM.pdf]

Supplementary Table S1: List of tyrosine kinase inhibitors used in this study

Target	Compound	Company
MET	AMG337	Selleck
	BMS-794833	MedChemExpress
	capmatinib	Selleck
	crizotinib	Selleck
	glumetinib	Selleck
	JNJ-38877605	MedChemExpress
	JNJ-38877618	Selleck
	MK-8033	Chem scene
	ningetinib	MedChemExpress
	PF-04217903	Drug libarary
	PHA-665752	Selleck
	SAR125844	MedChemExpress
	savolitinib	MedChemExpress
	SGX-523	MedChemExpress
	SU11274	Drug libarary
	S49076	Selleck
	tepotinib	Selleck
	TPX-0022	MedChemExpress
	AMG-458	SYN kinase
	altiratinib	Cayman Chemical
	BMS777607	Selleck
	BMS754807	Chemscene LLC
	cabozantinib	Selleck
	CEP-40783	CHEMGOOD
	foretinib	MedChemExpress
	glesatinib	Selleck
	golvatinib	MedChemExpress
	merestinib	Selleck
	sitravatinib	Selleck
	XL-092	MedChemExpress
	amuvatinib	Selleck
	tivantinib	Selleck
	MK-2461	Selleck

Target	Compound	Company
ALK	brigatinib	Selleck
	A83-01	Drug libarary
	LDN193189	Drug libarary
EGFR	BPIQ-II	Drug libarary
	AG1478	Drug libarary
	AG490	Drug libarary
	gefitinib	Drug libarary
	genistein	Drug libarary
	erlotinib	Drug libarary
	osimertinib	Selleck
	AG1478	Drug libarary
	AZ5104	Selleck
EGFR/HER2	AG825	Drug libarary
	afatinib	Selleck
	dacomitinib	Selleck
	poziotinib	Selleck
	lapatinib	Selleck
	pyrotinib	Selleck
HER2	neratinib	Selleck
	TAS0728	Selleck
	AG825	Drug libarary
	irbinitinib	Selleck
IGF-1R	picropodophyllin	Selleck
	linsitinib	Drug libarary
	AG1024	Drug libarary
	AGL 2263	Drug libarary
	OSI-906	Drug libarary
Bcr-Abl	AG957	Drug libarary
	nilotinib	Drug libarary
	dasatinib	Drug libarary
	imatinib mesylate	Drug libarary
FGFR	PD173074	Drug libarary
	SU4984	Drug libarary
	SU5402	Drug libarary
PDGFR	AG1296	Drug libarary
	SU11652	Drug libarary
	PDGF receptor TKI V	Drug libarary
	PDGF receptor TKI IV	Drug libarary
VEGFR	VEGFR receptor TKI II	Drug libarary
	VEGF recptor 2 KI I	Drug libarary
	SU1498	Drug libarary
TrKA	TrkA inhibitor	Drug libarary
Flt-3	Flt-3 Inhibitor	Drug libarary
Fms	cFMS receptor TKI	Drug libarary
Flk-1	SU1498	Drug libarary

Supplementary Table S2. List of antibodies used in this study

Target	Product name	Company	Code
Phospho-MET	Phospho-Met (Tyr1234/1235) Antibody	Cell Signaling Technology	#3126
MET	Met (D1C2) XP® Rabbit mAb #8198	Cell Signaling Technology	#8198
Phospho-AKT	Phospho-Akt (Ser473) (D9E) XP® Rabbit mAb	Cell Signaling Technology	#4060
AKT	Akt Antibody	Cell Signaling Technology	#9272
Phospho-ERK1/2	Phospho-p44/42 MAPK (Erk1/2) (Thr202/Tyr204) (D13.14.4E) XP® Rabbit mAb	Cell Signaling Technology	#4370
ERK1/2	p44/42 MAPK (Erk1/2) Antibody	Cell Signaling Technology	#9102
β-Actin	β-Actin (13E5) Rabbit mAb	Cell Signaling Technology	#4970
secondary antibody	secondary anti-rabbit IgG, HRP-linked antibody	Cell Signaling Technology	#7074

Supplementary Table S3. Results of drug screening other than tyrosine kinase inhibitors

Category	Compound	WT+IL3	Ex14skip	D1228A	D1228Y
blank	None (DMSO)				
antitumor (thymidylate synthetase)	5-FU		85.9	95.5	
antitumor (aminopeptidase B)	Bestatin		82.7	95.9	86.0
antitumor (DNA)	Bleomycin sulfate		76.6	58.3	41.9
antitumor (DNA)	Cisplatin		81.4	93.8	
antitumor (DHFR)	Methotrexate	3.2	4.0	7.3	15.8
antitumor (DNA)	Mitomycin C		71.9	53.1	31.6
antitumor (tubulin)	Vinblastine sulfate	2.7	3.2	3.6	14.6
antitumor (tubulin)	Paclitaxel	9.5	4.7	4.6	14.5
antitumor (AR)	Flutamide	97.4	74.5	90.2	72.1
antitumor (DNA)	Daunorubicin, HCl	3.6	3.4	9.3	12.0
antitumor (DNA)	Doxorubicin, HCl	5.7	3.8	13.9	13.4
antitumor (ER)	Tamoxifen, citrate	90.4	68.0	86.7	65.1
antitumor (RNA)	Actinomycin D	2.6	2.9	3.4	11.4
antitumor (topo I)	Camptothecin	6.4	3.9	4.5	13.1
antitumor (topo I/II)	Aclarubicin	71.8	85.8	87.4	41.3
antitumor (topo II)	Etoposide (VP-16)		72.5	79.1	52.9
actin filament	Cytochalasin D	90.8	50.4	54.9	30.7
adenylcyclase	2',5'-dideoxyadenosine	88.8	75.9	91.2	66.6
AKT	AKT inhibitor	89.1	67.9	89.1	59.2
AKT	NL-71-101	89.8	69.0	87.9	67.3
CAMKII	KN93	92.1	66.8	86.6	78.2
caspase	Z-VAD-FMK		81.5	91.5	74.6
CDC2	Kenpaullone	99.4	82.1	94.5	82.6
CDK2	Purvalanol A	90.6	76.4	92.4	72.8
CDK4	3-ATA	91.8	74.1	90.0	64.9
CDKs	Olomoucine	85.1	72.7	89.5	73.3
CKII	TBB	96.4	79.8	86.9	77.4
COX-1	Sulindac sulfide	89.3	70.8	87.2	69.1
COX-1	Valeryl salicylate	92.6	71.3		63.6
COX-2	NS-398	[illegible]	82.8	91.9	85.5
COX	Sodium salicylate		74.6	96.2	73.5
cyclicphosphodiesterase	Theophylline	95.5	66.9	88.1	76.7
DNA methyltransferase	Azacytidine	86.4	63.9	88.4	37.0
DNA polymerase	Aphidicolin		79.6	78.2	63.5
farnesyltransferase	Manumycin A	92.2	70.8	88.9	73.3
farnesyltransferase	FTI-276	97.8	74.0	96.0	77.5
geranylgeranyltransferase I	GGTI-286	92.7	69.2	87.8	66.7
GR	Dexamethasone	13.6	76.5	67.5	43.8
GSK-3	GSK-3 inhibitor II	93.8	77.7	88.2	68.8
HDAC	Scriptaid	93.8	63.4	73.6	38.7
HDAC	Trichostatin A	2.5	3.7	3.6	13.1
protein synthesis	Cycloheximide	53.2	35.1	36.3	33.9
HMG-CoA reductase	Lovastatin	91.7	84.2	97.5	92.7
HSP90	Radicicol	8.4	2.9	3.6	12.4
HSP90	17-AAG	95.1	90.7	39.0	19.4
iNOS	1400W, HCl	89.1	86.6	86.2	69.4
iNOS	AMT, HCl	89.3	68.1	90.3	65.0
Jak-2	AG490	98.3	71.9	86.9	69.0
Jak-2	Cucurbitacin I	16.9	7.8	8.2	16.6
JNK	SP600125	84.1	79.9	97.3	84.7
lck (p56), TYK	Damnacanthal	91.6	79.1	90.1	70.1
MEK	PD 98059	90.9	75.1	84.2	65.3
MEK	U0126	91.6	80.1	86.7	67.2
methionine aminopeptidase	Fumagillin	69.6	43.3	45.2	21.4
MMP	GM 6001	89.9	70.9	86.6	71.9
NF-kB	N-Acetyl-L-cysteine	91.8	70.0	88.4	75.0
NOS	Aminoguanidine, HCl	95.1	75.5	91.0	73.7
NOS	L-NMMA	95.2	94.8	98.3	85.5
p38 (MAPK)	PD169316	90.8	82.2	95.6	72.3
p38 (MAPK)	SB 203580	90.2	82.8	88.3	67.7

Category	Compound	WT+IL3	Ex14skip	D1228A	D1228Y
blank	None (DMSO)				
p70 S6K	Rapamycin	52.2	61.1	17.4	21.5
PARP	NU1025	87.3	75.1	88.2	74.1
PARP-1	Benzamide	91.5	76.4	90.3	73.6
PC-PLC	D609	90.2	71.7	89.2	77.0
PDE	IBMX		77.6	94.1	92.2
PDE (cAMP)	Ro-20-1724		90.5	95.8	84.1
PDE (cGMP)	Zaprinast		83.7	96.8	77.8
PI3K	LY294002	99.2	73.9	87.4	74.6
PI3K	Wortmannin	97.4	75.0	88.6	76.9
PKA	H-89, HCl	94.7	74.7	88.3	71.8
PKC	Bisindolymaleimide I, HCl	94.3	74.3	87.8	66.9
PKC, PKA	H-7		78.9	91.1	86.2
PKC, PKA, PKG, MLCK	Staurosporine	2.9	3.8	4.1	15.7
PLA2	cPLA2inhibitor	88.2	72.7	92.4	75.4
PLA2	OBAA	85.6	75.7	90.8	81.1
PP2A	Cantharidin	95.5	73.2	87.2	72.8
PP2A	Cytostatin	93.9	78.3	90.0	79.8
PP2B/cyclophilin	Cyclosporin A	95.8	68.6	89.8	83.1
PP2B/FKBP	FK-506	95.1	66.5	89.1	80.1
proteasome	MG-132	98.6	91.8	93.8	15.8
proteasome	Lactacystin	97.1	[illegible]	94.3	
ribonucleotide reductase	Hydroxyurea		98.1	98.4	91.3
ROCK	HA1077	[illegible]	88.3		76.7
ROCK	Y27632		82.5	91.2	73.1
Src, Fyn, Lck	PP1 (analog)		89.5	96.5	71.0
Src, Fyn, Lck	PP-H		85.9	95.6	83.3
tubulin depolymerization	Nocodazole	5.6	4.3	5.7	15.6
tyr phosphatase (PTP)	Dephostatin		91.1		94.2
AK	ABT-702	74.7	90.9	91.1	86.0
AKT	Akt Inhibitor IV	20.0	24.4	77.1	
AKT	Akt Inhibitor VIII, Isozyme-Selective, Akti-1/2	46.2	81.4	87.9	81.8
AKT	Akt Inhibitor XI	51.2	85.3	89.4	69.7
AMPK	compound C	53.7	85.3	91.5	
ATM	ATM/ATR kinase inhibitor	71.2	87.4	91.6	83.1
ATM	ATM kinase inhibitor	92.1	97.9	92.8	82.1
Aurora	Aurora kinase/cdk inhibitor	90.0	95.9	91.8	97.6
Aurora	Aurora kinase inhibitor II	81.8	73.3	83.7	75.6
Aurora	Aurora kinase inhibitor III	79.7	71.4	85.0	75.4
BTK	LFM-A13	75.1	70.9	84.0	68.2
BTK	Terreic acid	64.9	72.3	83.4	82.4
CAMKII	KN-93	68.2	76.4	84.9	65.9
CAMKII	KN-62	91.9	84.7	87.4	66.6
CAMKII	Lavendustin C	95.1	77.6	93.5	84.8
CDK	Kenpaullone	79.2	74.1	83.4	65.5
CDK	purvalanol A	82.0	70.3	86.0	68.5
CDK	Olomoucine	82.3	67.2	85.7	70.6
CDK	Alsterpaullone, 2-cyanoethyl	64.3	54.5	74.8	53.8
CDK	Cdk1/2 inhibitor III	8.9	11.3	7.2	14.4
CDK	Cdk2/9 inhibitor	72.2	61.8	80.3	62.0
CDK	NU6102	91.4	86.7	89.3	76.5
CDK	Cdk4 inhibitor	94.6	77.5	97.8	78.1
CDK	NSC625987	79.7	72.8	96.0	70.3
Chk	SB218078	55.7	38.6	38.8	18.2
Chk	isogranulatimide	83.6	66.5	91.8	80.3
Chk	Chk2 inhibitor	78.7	68.1	83.2	70.0
Chk	Chk2 inhibitor II	77.7	69.8	85.7	70.4
CK	Ellagic acid	79.4	70.0	82.4	70.0
CK	TBB	89.8	85.0	84.9	76.0
CK	DMAT	94.8	80.5	93.3	97.7
CK	D4476	85.1	66.6	83.6	76.6

Inhibitory rate of cell growth to DMSO (%)

0 50 100

Supplementary Table S3. (continued) Results of drug screening other than tyrosine kinase inhibitors

Category	Compound	WT+IL3	Ex14skip	D1228A	D1228Y
blank	None (DMSO)				
Clk	TG003	87.2	70.9	92.2	61.9
DGK	Diacylglycerol kinase inhibitor II	79.5	69.6	96.2	66.7
DNA-PK	IC60211	79.5	67.0	83.0	62.1
eEF2	TX-1918	80.2	73.2	83.7	76.3
Fyn	SU6656	81.3	66.6	82.1	[illegible]
GSK	GSK-3 inhibitor IX	72.7	69.8	77.3	73.7
GSK	1-Azakenpaullone	91.9	84.7	82.5	75.5
GSK	indirubin-3'-monoxime	92.7	82.9	92.7	89.4
IKK	BMS-345541	82.0	74.3	82.1	65.0
IKK	IKK-2 inhibitor VI	81.9	74.0	81.0	29.0
IRAK	IRAK-1/4 inhibitor	83.9	70.3	83.8	61.2
Jak	JAK Inhibitor I	90.9	87.2	90.7	60.3
Jak	JAK3 Inhibitor VI	94.3	99.5	97.0	75.6
JNK	SP600125	77.5	70.8	98.9	78.9
JNK	JNK inhibitor VIII	80.5	66.7	80.7	80.0
Lck	Damnacanthal	81.7	63.2	86.9	70.4
Lck	PP2	85.4	72.6	82.8	71.3
MAPK	ERK inhibitor II	82.4	70.3	84.7	71.0
MEK	PD98059	84.7	70.0	82.6	68.1
MEK	U-0126	93.5	89.5	89.7	73.9
MEK	MEK inhibitor I	95.1	85.8	91.7	87.8
MLCK	ML-7	80.0	70.2	94.8	68.8
p38	SB202190	83.1	72.8	98.0	73.4
p38	SB239063	80.6	79.1	[illegible]	78.4
PI3K	LY-294002	86.7	73.4	96.5	81.9
PI3K	Wortmannin	79.5	73.8	84.2	82.3
PKA	H-89	81.6	72.8	84.9	84.6
PKA	4-cyano-3-methylisoquinoline	85.5	71.2	88.1	69.1
PKC	Bisindolymaleimide I, HCl	81.1	70.9	82.7	53.1
PKC	Go7874	87.2	73.9	81.3	36.8
PKG	Rp-8-CPT-cGMPS	96.3	87.1	91.4	87.8
PKG	KT5823	98.6	91.1	96.7	73.2
PKR	PKR inhibitor	66.8	69.4	88.1	75.9
Raf	RAF1 kinase inhibitor I	82.4	74.9	86.5	84.3
Raf	ZM 336372	81.6	76.9	97.6	91.1
ROCK	H-1152	85.9	79.7	90.6	88.5
ROCK	Y-27632	81.3	78.5	87.6	73.1
Hsp90	radicicol	7.1	2.9	3.1	13.2
Src	PP1 analog	[illegible]	93.4	91.3	88.1
Syk	Syk inhibitor	92.4	99.2	96.2	78.8
TGF-βRI	SB431542	95.7	93.9	96.1	86.6
TGF-βRI	TGF-b RI kinase inhibitor II	95.8	[illegible]	97.2	[illegible]
Tpl2	Tpl2 kinase inhibitor	91.7	87.8	90.5	[illegible]
mTOR	temsirolimus	46.4	75.1	25.2	17.1
HDAC	vorinostat	[illegible]	79.9	88.1	31.8
Proteasome	bortezomib	2.5	4.3	4.6	9.3
mTOR	everolimus	47.0	72.1	23.4	17.1
Rho/SRF	CCG-1423	85.9	71.0	[illegible]	53.4
PIM	PIM1/2 Kinase Inhibitor V	96.5	80.4	[illegible]	60.1
PIM	PIM1 Inhibitor II	[illegible]	92.3	[illegible]	65.9
Hedgehog	AY 9944	87.7	67.4	[illegible]	52.4
Hedgehog	cyclopamine	82.5	76.5	[illegible]	58.4
Hedgehog	Jervine	86.5	73.6	[illegible]	68.9
STAT3	WP1066	88.8	74.1	[illegible]	50.9
STAT3	5,15-DPP	90.2	71.7	[illegible]	71.5
Wnt	IWP-2	89.8	76.7	[illegible]	59.4
Wnt	IWR-1-endo	93.7	74.7	[illegible]	51.2
Wnt	FH535	[illegible]	86.4	[illegible]	73.4
Notch	DAPT	92.5	70.3	[illegible]	51.6
tankyrase-selective PARP	XAV939	83.8	67.2	[illegible]	51.5

Category	Compound	WT+IL3	Ex14skip	D1228A	D1228Y
blank	None (DMSO)				
pan-PARP	PJ-34	87.0	71.1	[illegible]	64.3
PARP-1/2-selective	Olaparib	91.2	73.9	[illegible]	43.9
antipsychotic drug	chlorpromazine hydrochloride	92.2	73.9	[illegible]	59.1
depression treatment	desipramine hydrochloride	90.6	74.2	[illegible]	67.1
golgi inhibitor	brefeldin A	97.3	76.1	[illegible]	68.0
stress inducer	anisomycin	3.0	3.4	5.7	10.0
thalidomide family	thalidomide	85.1	68.9	[illegible]	63.8
thalidomide family	lenalidomide	93.2	77.6	[illegible]	64.4
retinoids	tretinoin	88.4	61.1	82.5	49.4
retinoids	tamibarotene	88.4	61.1	80.2	42.7
DNA alkylation	temozolomide	94.3	74.7	[illegible]	65.0
mTOR	Torkinib	[illegible]	83.0	[illegible]	54.2
lipase	orlistat	[illegible]	89.3	[illegible]	68.0
AR	MDV3100	88.1	66.9	98.0	60.2
caspase activator	PAC-1	94.6	66.1	[illegible]	53.1
blc-2	ABT-737	80.8	69.2	97.0	62.4
G9a	UNC0638	95.1	67.1	[illegible]	49.2
G9a	BIX01294	90.8	70.7	98.0	59.7
LSD1	S2101 (LSD1 inhibitor II)	93.6	72.8	[illegible]	59.4
PRMT1	AMI-1	[illegible]	77.2	[illegible]	70.4
p300	C646	[illegible]	80.0	[illegible]	74.1
SIRT1	SIRT1 inhibitor III	90.9	69.4	[illegible]	63.0
SIRT1/2	Tenovin-6	88.9	66.7	[illegible]	56.5
HDAC8	PCI-34051	90.6	74.8	[illegible]	60.5
BRD4 bromodomain	(+)-JQ1	12.5	17.6	14.9	10.5
Telomerase	TMPyP4	91.8	74.8	[illegible]	51.2
PARP	BSI-201 (Iniparib)	91.8	70.4	[illegible]	71.0
PARP	ABT-888 (Veliparib)	[illegible]	80.5	[illegible]	67.1
PARP	AG014699 (Rucaparib)	[illegible]	82.2	[illegible]	62.4
PARP	MK-4827 (Niraparib)	95.2	73.9	[illegible]	50.7
Aurora	ENMD-2076	94.1	64.2	[illegible]	64.2
Aurora	MLN8237	62.6	17.4	16.9	12.7
Survivin	YM155	92.4	75.6	[illegible]	60.4
PDK1	OSU-03012	94.2	77.0	[illegible]	70.9
DNMT	Decitabine	98.7	57.9	74.3	20.5
BRAF	Vemurafenib	94.3	69.6	[illegible]	59.7
JAK	Ruxolitinib	95.1	70.0	[illegible]	69.1
Hedgehog	Vismodegib	[illegible]	78.5	[illegible]	67.4
GLI1	Gant61	93.6	69.1	[illegible]	57.6
GSK-3	BIO	81.2	75.7	96.7	70.0
GSK-3	TWS119	93.5	70.5	[illegible]	58.2
GSK-3	CT99021	88.4	80.8	[illegible]	59.9
TGFb-R	LY2157299	98.5	74.3	[illegible]	63.3
TGFb-R	SD208	95.8	79.3	[illegible]	53.5
ROCK	Thiazovivin	[illegible]	84.9	[illegible]	73.3

Inhibitory rate of cell growth to DMSO (%)

0 50 100

Supplementary Table S4. Type II MET-TKIs evaluated in this study

Compound	Developer	Target	Clinical developmental status	Dose	Cmax	Ref
**merestinib (LY2801653)**	Eli Lilly	MST1R, AXL, ROS1, MKNK1/2, FLT3, MERTK, DDR1/2	Phase II (NCT02920996) Ongoing	120mg/day	539nM	Hwang, Jimmy, et al. AACR. 2014
**cabozantinib (XL184)**	Exelixis	VEGFR2, c-Met, Ret, Kit, Flt1/3/4, Tie2, and AXL	Phase II (NCT03911193) Ongoing	60mg/day	900nM*	kurzrock,R, et al. J clin Oncol. 2011
**altiratinib (DCC-2701)**	Deciphera	MET, TIE2, VEGFR2, FLT3, Trk1/2/3	Terminated	N/A		
**foretinib (XL880, GSK1363089)**	Exelixis	MET, VEGFR, KDR	Terminated	240mg, for 5days every 2 weeks	340nM	Joseph Paul Eder, et al. Clin Cancer Res. 2010
				80mg/day	72.2nM	Geoffrey I. Sahpiro, et al. Invest New Drugs. 2013
**sitravatinib (MGCD-516)**	Mirati Therapeutics, Inc.	MET, AXL, MER, VEGFR1/2/3, KIT, FLT3, DDR1/2, TRK A/B	Ongoing	120mg/day	115.14nN	Pavlos Msaouel, et al. SITC 2019
**CEP-40783 (RXDX-106)**	Daiichi Sankyo /Teva Pharmaceutical Industries	MET, TAM (TYRO3, AXL, MER)	Terminated	N/A		

* The value was estimated from each Cmax reported at 80 mg/day and 40 mg/day respectively.

Supplementary Table S5. Clinical information of two phase I studies of foretinib

Trial No	Cancer type	Location	Patients number	MET selection	ORR (%)	DCR (%)	MTD	DLT	Common AE	RP2D	Ref
NCT00742131	Solid tumors	**US**	40<br>(n=0, lung cancer)	(-)	7.5%<br>(3/40)	62.5%<br>(25/40)	3.6mg/kg	Increased AST<br>Increased lipase	Hypertension<br>Fatigue<br>Diarrhea<br>Vomiting<br>Proteinuria<br>Hematuria	240mg,<br>for 5days every 2 weeks	[33]
NCT00743067	Solid tumors	**US**	37<br>(n=4, lung cancer)	(-)	0%	74%<br>(24/31)	80mg	Hypertension<br>Dehydration<br>Diarrhea	Fatigue,<br>Hypertension,<br>Nausea,<br>Diarrhea	80mg/day	[34]

Abbreviations: ORR; Objective response rate, DCR; Disease control rate, MTD; Maximum tolerated dose, DLT; Dose limiting toxicity, AE; Adverse event, RP2D; Recommended phase 2 dose

Supplementary Table S6. Clinical information of five phase II studies of foretinib

No	Cancer type	Location	Prior treatment	Patients number	Dose	MET selection	Cohort / subgroup analysis	ORR (%)	DCR (%)	mPFS (month)	mOS (month)	Ref
1	Hepatocellular carcinoma	Global (Asia)	(-)	35	30mg/day	(-)	All patients	22.9% (8/35)	82.9% (29/35)	4.2	15.7	[35]
2	Breast cancer	Canada	(+)	45	60mg/day	(-)	All patients (MET amp ; n=0)	4.7% (2/43)	46% (17/37)	N/A		[36]
3	Squamous Cell Cancer of the Head and Neck	US	(+)	41	240mg, for 5 days every 2 weeks	(-)	All patients	0% (0/14)	50% (7/14)	3.65 [3.4-5.3]	5.59 [3.71-NA]	[37]
4	Gastric Cancer	US		74	・cohort A : 240mg, for 5 days every 2 weeks ・cohort B: 80mg/day	(-)	All patients	0% (0/71)	23% (10/71)			[27]
							MET amplified (MET/CEP7 ratio > 2.0)	0% (0/3)	33% (1/3)			
5	Papillary Renal-Cell Carcinoma	US	(+)	74	・cohort A : 240mg, for 5 days every 2 weeks ・cohort B : 80mg/day	(-)	All patients	13.5% (10/74)		9.3 [6.9-12.9]	Not reached	[26]
							MET germline mutation (+) (n=10)	50% (5/10)	100% (10/10)			
							MET germline mutaiton (-) (n=57)	8.7% (5/57)				

Abbreviations: ORR; Objective response rate, DCR; Disease control rate, mPFS; median progressive free survival, mOS; median overall survival
