# Supplementary material for: Foretinib can overcome common on-target resistance mutations after capmatinib/tepotinib treatment in NSCLCs with MET exon 14 skipping mutation
Source: J Hematol Oncol. 2022 Jun 11;15:79. doi: 10.1186/s13045-022-01299-z (PMC9188708; doi:10.1186/s13045-022-01299-z)
Supplement: Supplementary file 2 — Additional file 2: Fig. S1. Drug screening against MET D1228A/Y using a drug library. Fig. S2. Summary of growth inhibitory curves of eight MET-TKIs for Ba/F3 cells with each MET mutation. Fig. S3. Evaluation of the effects of secondary mutations on sensitivity to MET-TKIs using the Hs746t cell line. Fig. S4. Analysis of the secondary resistance mutations against 1st line foretinib. Fig. S5. Growth inhibition curves for ENU-derived foretinib resistant Ba/F3 cells carrying MET secondary mutation. Fig. S6. Comparison of the structures of each type II MET-TKI. Fig. S7. Summary of growth inhibitory curves of three MET-TKIs for Ba/F3 cells with MET tertiary mutations. Fig. S8. Structure of MET in the DFG-in and DFG-out conformations. [file 13045_2022_1299_MOESM2_ESM.pdf]

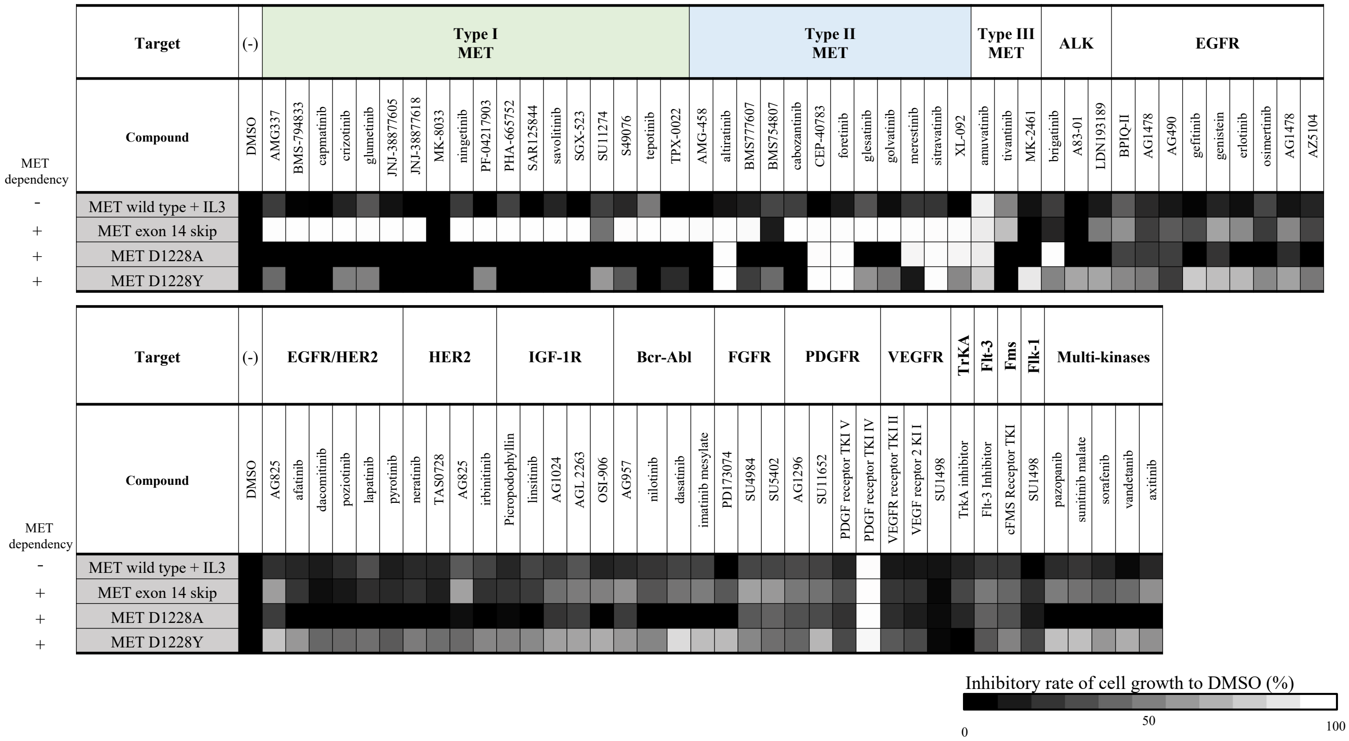

Supplementary Figure S1.

Drug screening against MET D1228A/Y using a drug library. The activities of 300 drugs at 100 nM were evaluated against 4 types of Ba/F3 cells: IL3-dependent Ba/F3 cells expressing wild-type *MET* and IL3-independent Ba/F3 cells expressing *MET* with exon 14 skipping mutation (*MET*Δex14), *MET*Δex14 plus *MET* D1228A or *MET*Δex14 plus D1228Y. A total of 3×10^3 cells were seeded in each well of 96-well plates. After 24 hours, drugs were added at 100 nM. After a 72-hour incubation period, the absorbance at 450 nm was read after the addition of Cell Counting Kit-8 solution to each well. Cell viability (%) was evaluated and compared with the viability of cells treated with DMSO and is expressed in a heatmap based on the indicated colors. The activities of other drugs used in this screening are shown in Supplementary Table S3.

A

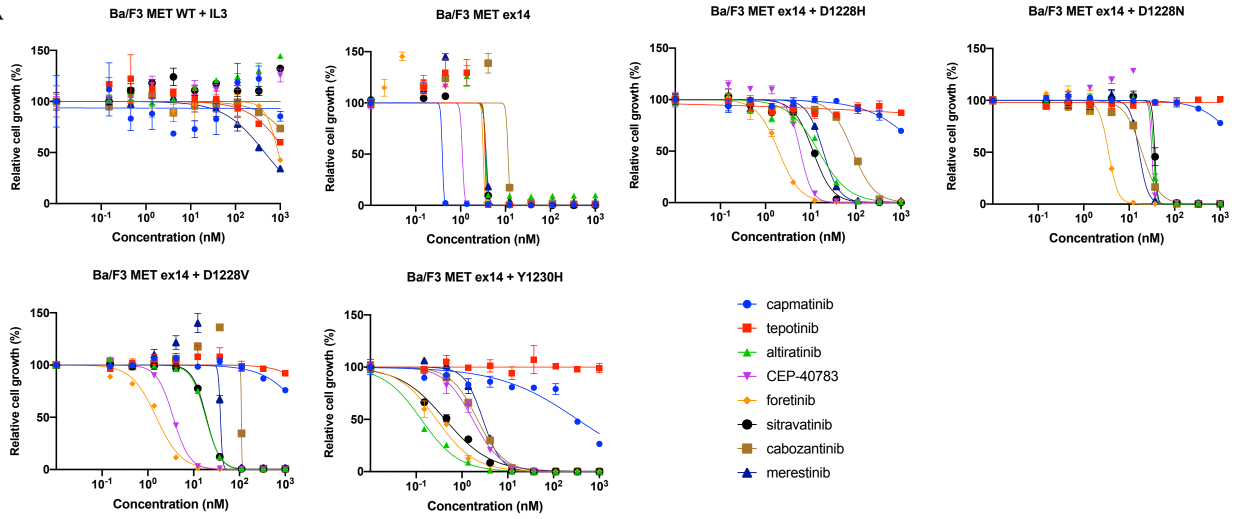

B

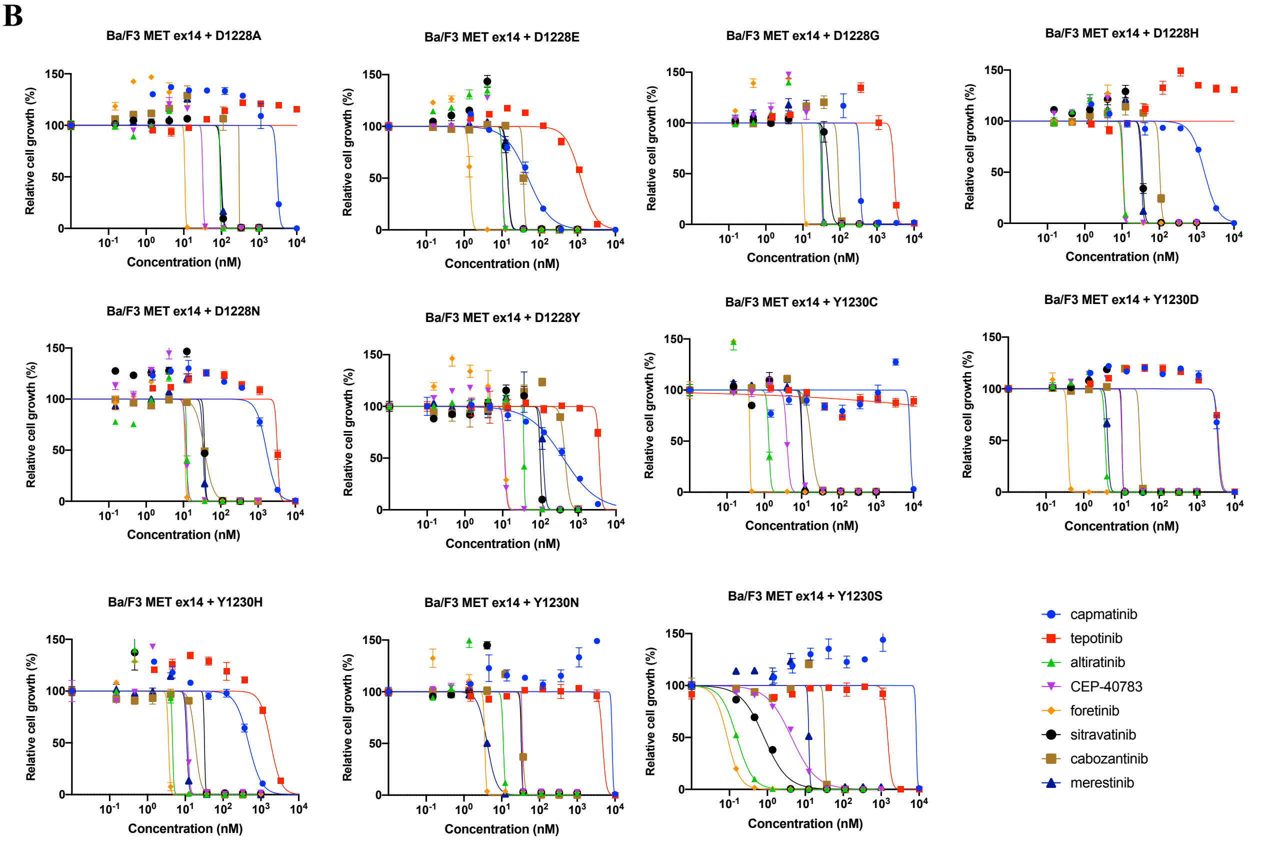

C

Method	cell line	mutation	capmatinib	tepotinib	altiratinib	CEP-40783	foretinib	sitravatinib	cabozantinib	merestinib
ENU derived	Ba/F3	**D1228H**	>1000	>1000	11	11	10	35	103	33
	Ba/F3	**D1228N**	>1000	>1000	12	12	11	37	37	34
	Ba/F3	**Y1230H**	470	>1000	4.6	12	3.6	32	18	11
Construct derived	Ba/F3	**D1228H**	>1000	>1000	15	5.9	2.0	11	85	19
	Ba/F3	**D1228N**	>1000	>1000	34	32	3.5	37	19	17
	Ba/F3	**Y1230H**	332.8	>1000	0.1	1.7	0.3	0.4	2.2	2.9

Supplementary Figure S2.
Growth inhibitory curves of eight MET-TKIs for Ba/F3 cells carrying the indicated *MET* mutations. (A) Ba/F3 clones established from the construct. **(B)** Ba/F3 clones derived from ENU mutagenesis. IC_{50} values are summarized in Figure 2A. **(C)** Comparison of IC_{50} values (nM) of each MET-TKI for ENU derived and construct derived clones

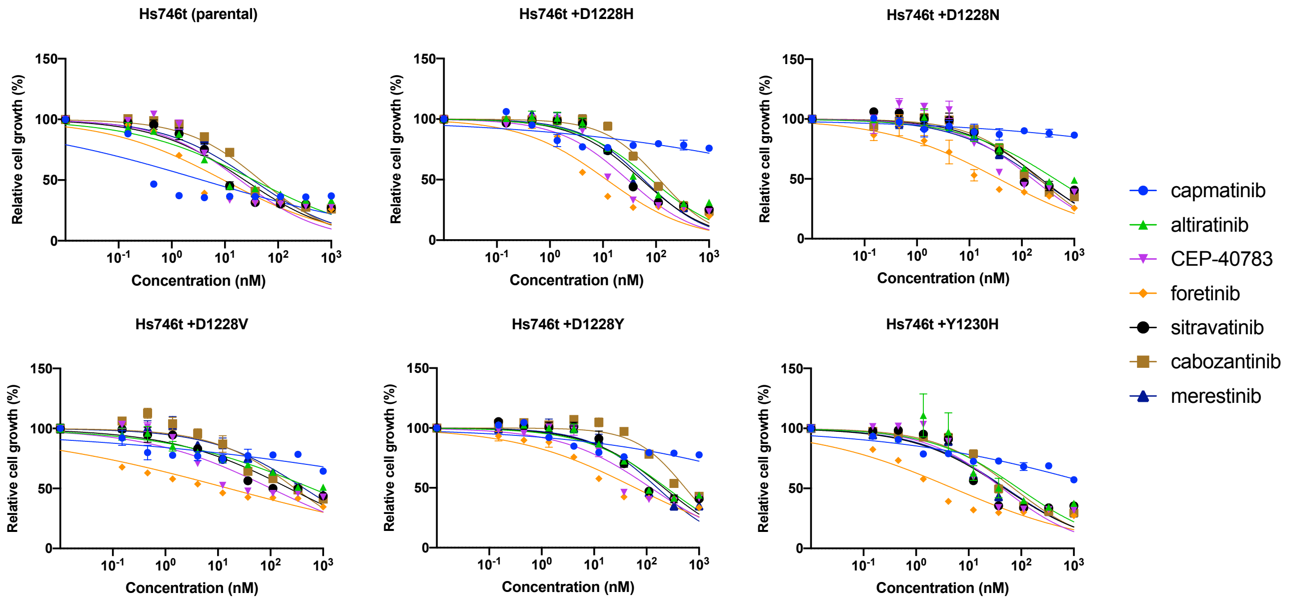

Supplementary Figure S3.

Evaluation of the effects of secondary mutations on sensitivity to MET-TKIs using the Hs746t cell line. Growth inhibition curves of the indicated MET-TKIs for Hs746t cells transduced with the indicated *MET* mutants. IC_{50} values are shown in Figure 3A.

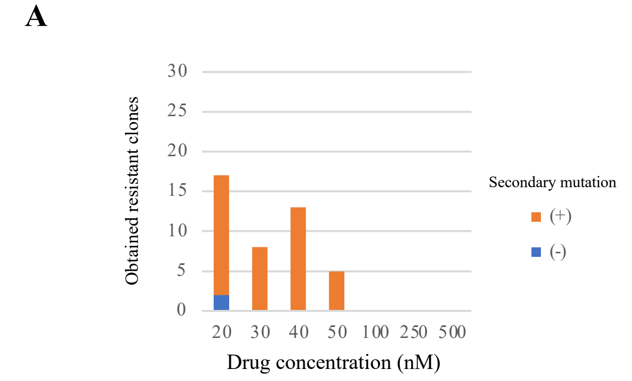

C

IC50 (nM)	capmatinib	tepotinib	foretinib
G1163R	3.1	53	59
L1195F	0.4	4	100
L1195V	9.9	43	10
F1200I	5.1	38	91
F1200L	0.7	11	29
F1200V	2.2	16	19
M1211L	1.1	3.9	31
G1163R+L1195F	1.3	67	39

$IC_{50} \leq 50nM$ | $50 < IC_{50} < 200nM$

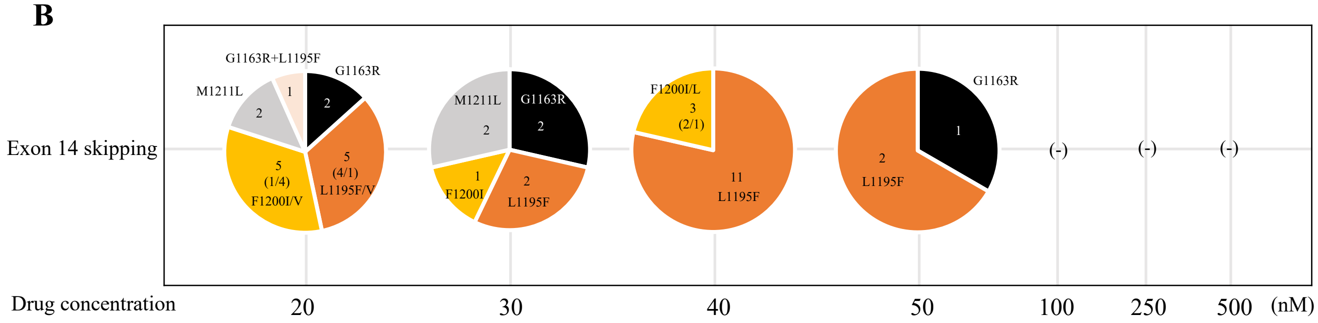

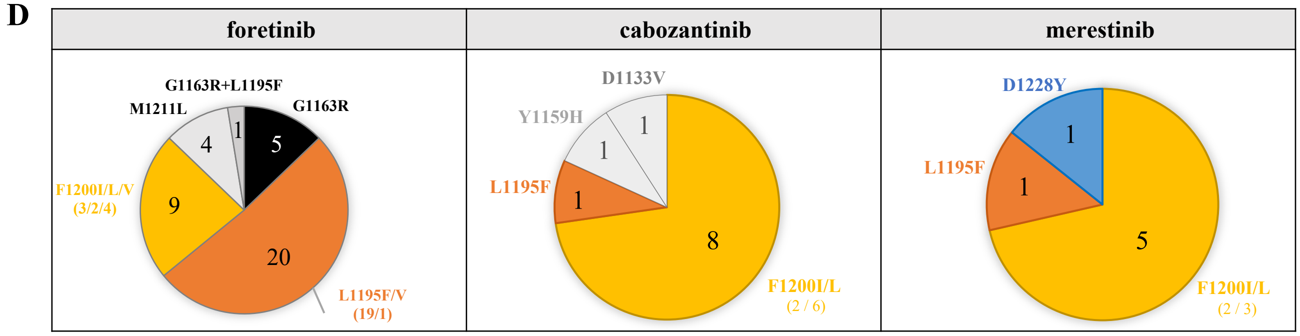

Supplementary Figure S4.

Analysis of the secondary resistance mutations against 1st line foretinib. (A) The number of obtained resistant clones and the percentage of clones that carried secondary mutations are shown for each drug concentration. Those with a secondary mutation are shown in orange, and those without are shown in blue. **(B)** The breakdown of the secondary mutations obtained is shown for each drug concentration. **(C)** The MTT assay was performed against the obtained resistant clones obtained by ENU mutagenesis, and the change in IC_{50} values for foretinib was evaluated. IC_{50} values are classified based on the indicated colors. They were also evaluated for the presence of cross-resistance to capmatinib and tepotinib. Growth inhibition curves are shown in Supplementary Figure S5. **(D)** Comparison of the types of resistant secondary mutations obtained by ENU mutagenesis of foretinib, cabozantinib, and merestinib. Data for cabozantinib and merestinib were taken from our previous report (Fujino, et al. 2019. JTO reference no. 16).

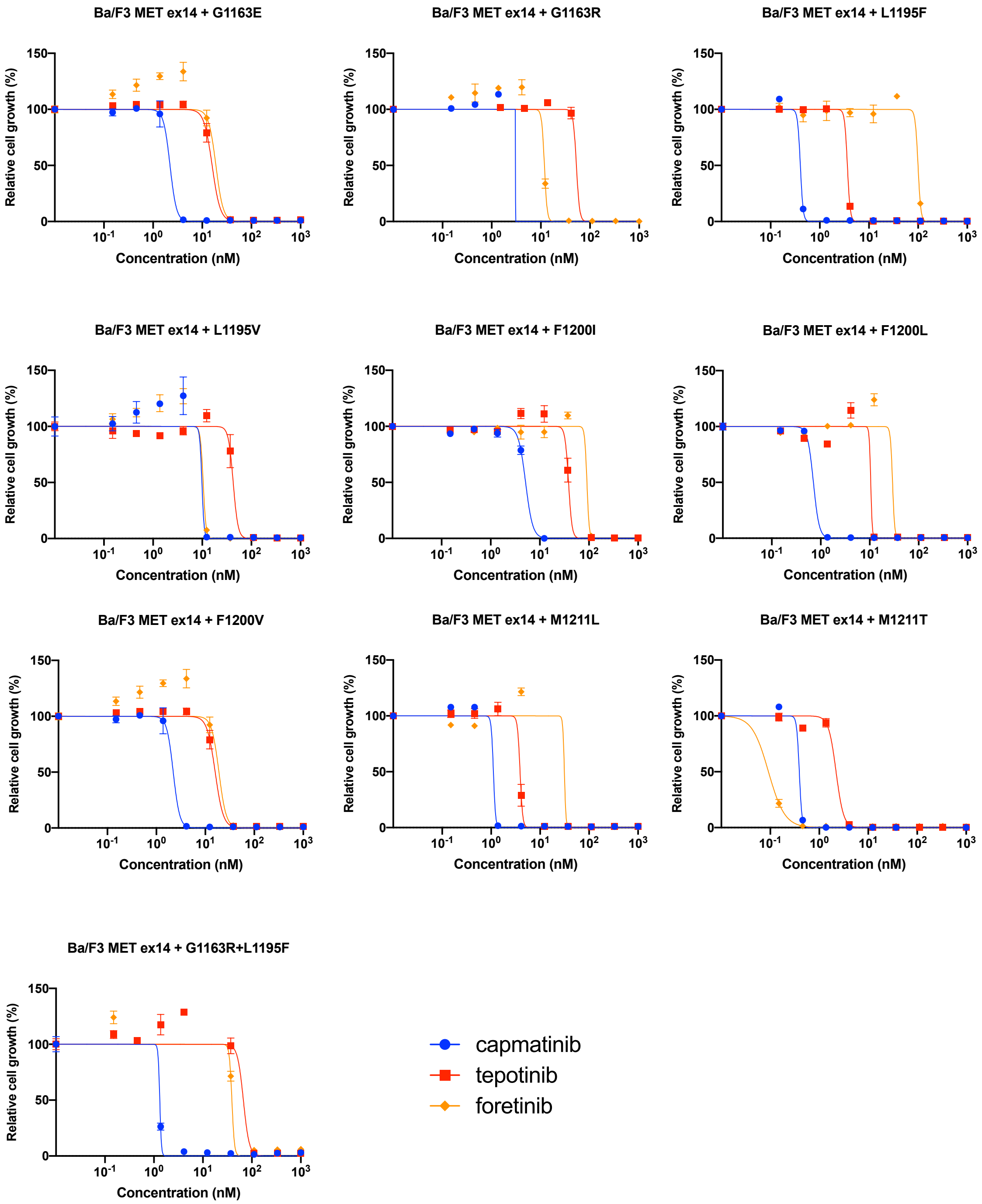

Supplementary Figure S5.
Growth inhibition curves for ENU-derived foretinib resistant Ba/F3 cells carrying *MET* secondary mutation.

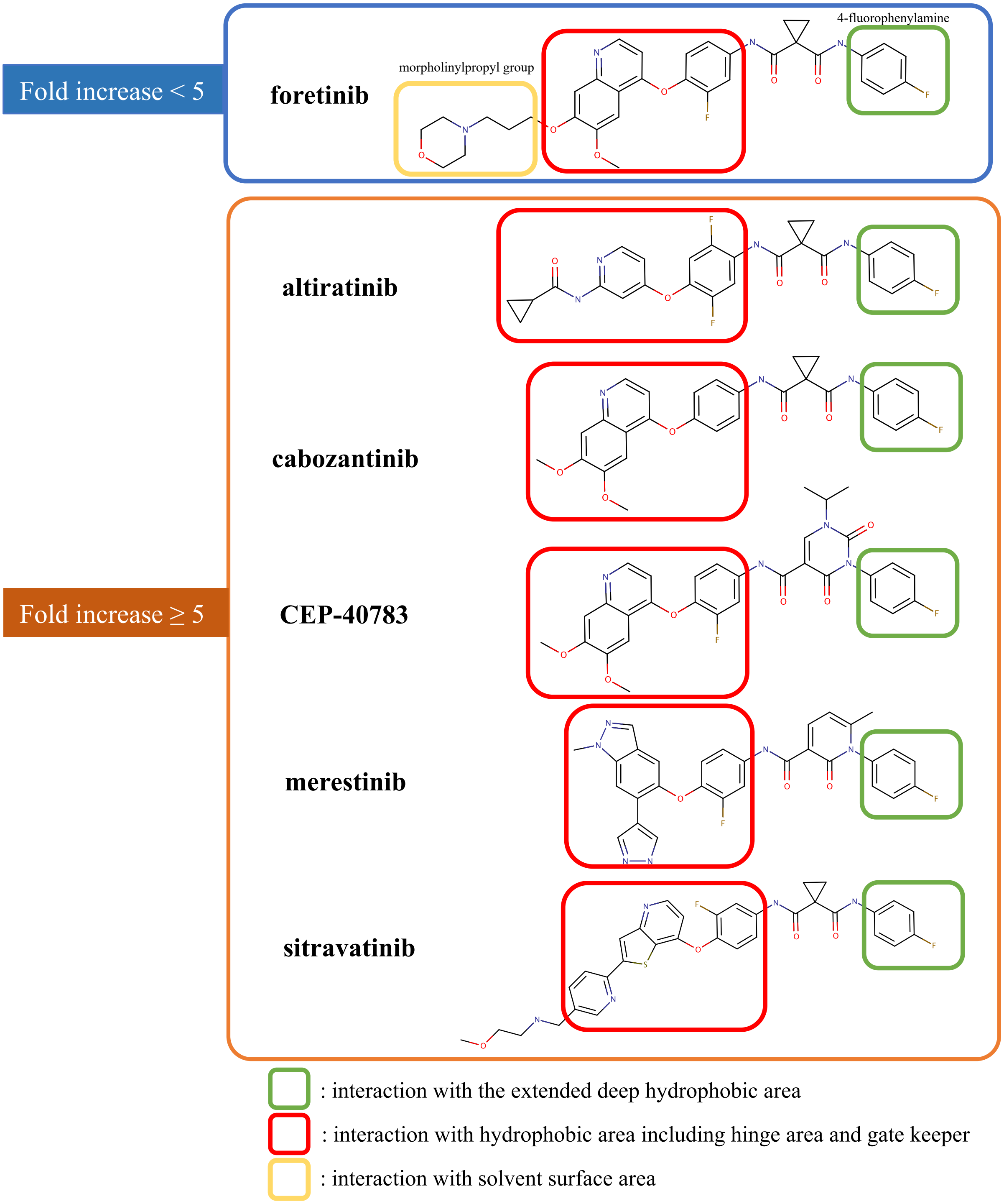

Supplementary Figure S6.
Comparison of the structures of each type II MET-TKI.

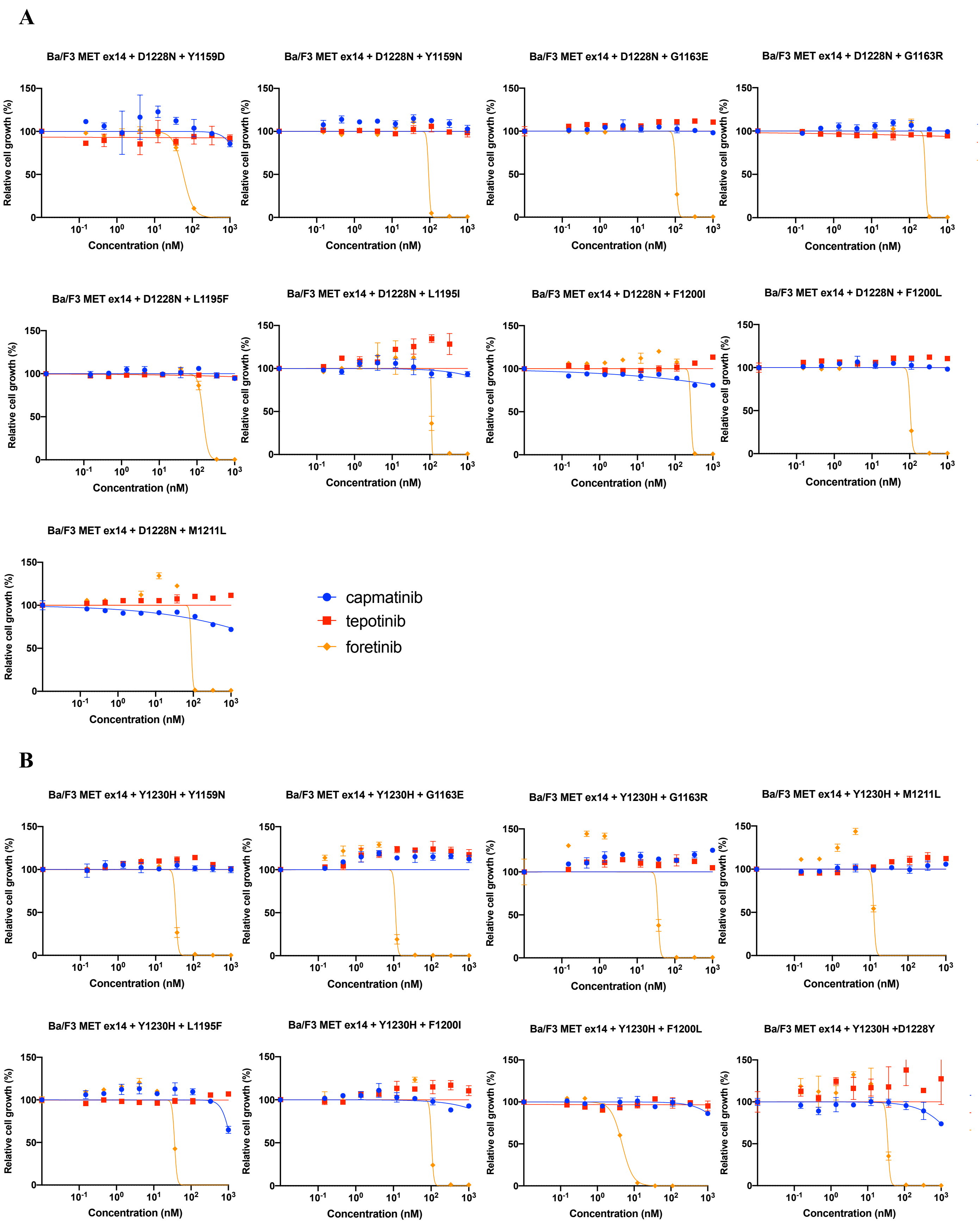

Supplementary Figure S7.

Growth inhibitory curves for ENU-derived foretinib resistant Ba/F3 cells carrying *MET* tertiary mutations. Each clone was established from Ba/F3 cells carrying *MET* exon 14 skipping mutation plus D1228N **(A)** or Y1230H **(B)**.

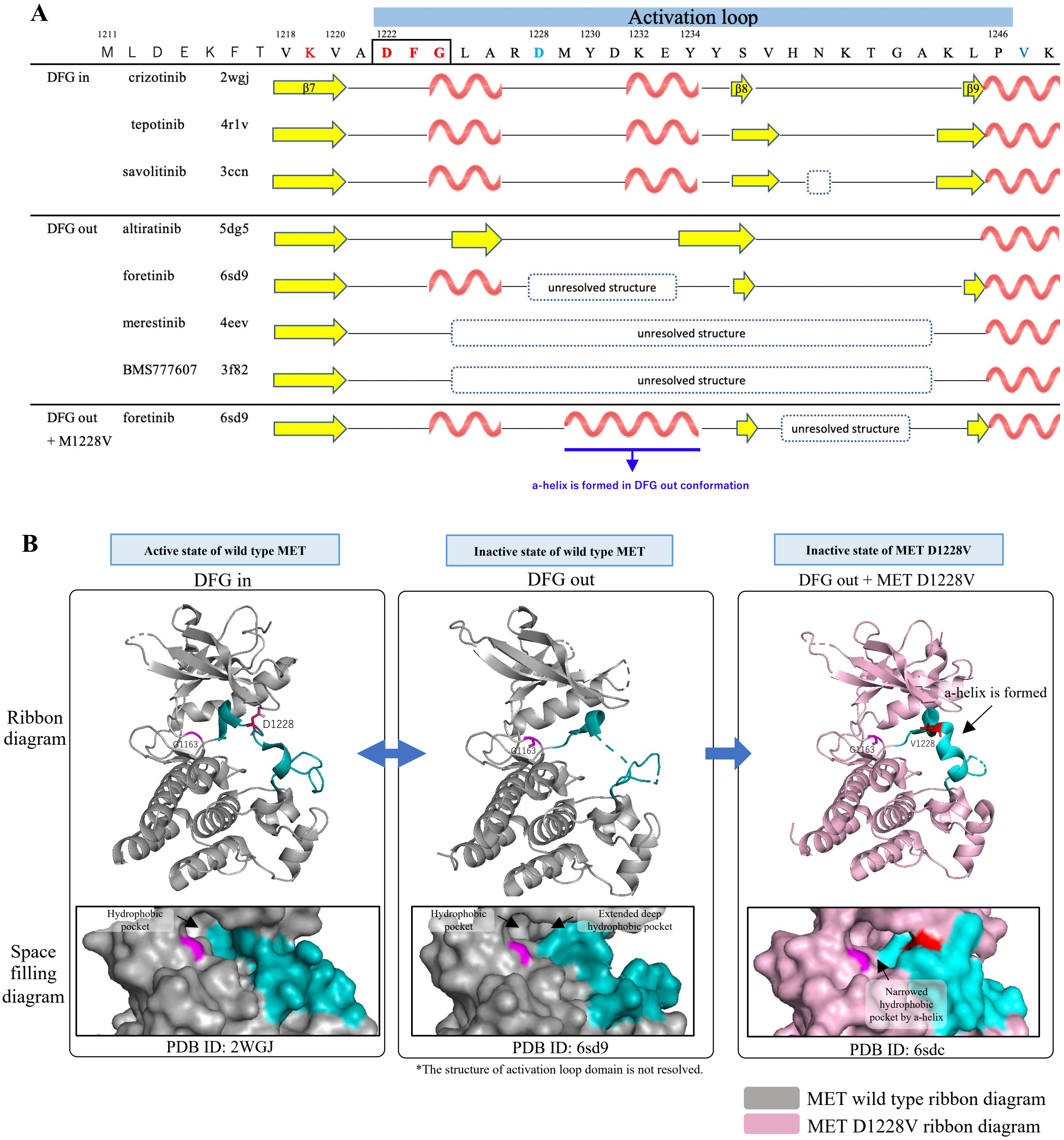

Supplementary Figure S8.

Comparison of structure of MET in the DFG-in and DFG-out conformations. (A) Schematic of the determined secondary structures of the DFG-in and DFG-out models from the deposited file. **(B)** In the activation loop region, which contains the DFG motif, D1228 forms constitutes an ATP-binding pocket in the DFG-in conformation. On the other hand, in the DFG-out conformation, the activation loop is thought to adopt a fluctuating structure, and the structure is often undetermined. However, when MET aspartic acid (D) 1228 was mutated to valine (V), an alpha-helical structure fixed near the entrance of the hydrophobic pocket formed.
